# Development of an instrument (Cost-IS) to estimate costs of implementation strategies for digital health solutions: a modified e-Delphi study

**DOI:** 10.1186/s13012-025-01423-w

**Published:** 2025-03-07

**Authors:** Thomasina Donovan, Bridget Abell, Steven M. McPhail, Hannah E. Carter

**Affiliations:** 1 Australian Centre for Health Services Innovation and Centre for Healthcare Transformation, School of Public Health and Social Work, Faculty of Health, Queensland University of Technology, Brisbane, QLD Australia; 2 Digital Health and Informatics, Metro South Health, Brisbane, QLD Australia

**Keywords:** Implementation costs, Implementation science, Health economics, Digital health

## Abstract

**Background:**

It is important to determine the relative value of health innovations when allocating limited healthcare resources. Implementation strategies require and consume healthcare resources yet are often excluded from published economic evaluations. This paper reports on the development of a pragmatic implementation costing instrument to assist with the planning, delivery, and evaluation of digital health implementation strategies.

**Methods:**

A modified e-Delphi process was adopted to develop an implementation costing instrument. Purposive sampling was used to recruit a panel of experts in implementation science, health economic evaluations and/or digital health from the academic, government, clinical or health service sectors. In each round, participants were sent an electronic questionnaire and a prototype of the implementation costing instrument. The prototype in the initial round was informed from a literature review and qualitative interview findings. The prototype was updated iteratively between rounds in response to the panel’s feedback. In subsequent rounds, participants also received the anonymous results of items that did not reach consensus in the previous round. Termination occurred once consensus was reached on integral questions (those pertaining specifically to the instrument design) or when three rounds were completed, to prevent sample fatigue. Consensus was defined as at least 75% of experts in agreement for any item.

**Results:**

Consensus was reached on the core components and design of the instrument from a panel of twelve experts in implementation science, health economic evaluations and/or digital health. Areas where consensus was not reached included users’ level of implementation science knowledge, specificity of the tool to digital health and accessibility via digital formats.

**Conclusions:**

Cost-IS is a pragmatic data collection instrument designed to estimate the costs of implementation strategies for digital health solutions. Further piloting of Cost-IS is required to establish its feasibility and generalisability.

**Supplementary Information:**

The online version contains supplementary material available at 10.1186/s13012-025-01423-w.

Contributions to the literature

- Cost-IS is a data collection instrument designed to assist with costing implementation strategies for digital health solutions.
- The development of Cost-IS involved a literature review, qualitative interviews with multidisciplinary experts, and a modified e-Delphi process.
- Rigorous costing of implementation strategies is important to support decision making and address a key knowledge gap in implementation science research.
- Further piloting of Cost-IS is required to determine its feasibility and generalisability.

## Introduction

Implementation strategies promote successful and sustainable implementation of healthcare interventions including digital health solutions [1]. Delivery of these implementation strategies typically requires appropriate resourcing [2]. Yet, costs associated with implementation strategies have been under reported in the literature, and often excluded from economic evaluations even those conducted in the field of improvement and implementation science [3, 4]. This represents a considerable challenge that undermines efforts to appropriately allocate healthcare-related resources for the implementation of healthcare models, services, or interventions [5]. A lack of data on implementation strategy costs poses a challenge to the implementation, sustainment and replication of digital health solutions [4]. Challenges to costing implementation strategies include inconsistencies in the conception of what constitutes implementation costs, a lack of cost data on financial allocation relating to organisational budgets, and a lack of methodological guidance. This is particularly true for implementation approaches in non-traditional or inter-disciplinary fields of practice, including digital health [6].

The cost of implementing new strategies (COINS) is one of few approaches for costing implementation strategies, however none have been designed for the unique context of implementing digital health solutions [6]. Digital health solutions include a wide array of technologies, including virtual care platforms, electronic reminder systems, computerised decision support systems, among others, that have potential to support better value through many possible vectors of service improvement opportunities. This may include improving accessibility, reducing human error, facilitating care coordination, improving practice efficiency, among other potential benefits [7]. The use of digital health solutions is increasing but the complexity of health systems presents challenges for their implementation [8]. The costs and cost-effectiveness of digital health interventions have been investigated [7], but often without considering the costs associated with implementation strategies [2, 6].

Implementation costs have been inconsistently defined across fields that do not share a common language, with digital health, implementation science and health economics being important exemplars of this phenomenon [6]. The boundaries for identifying implementation-related resources, separate from direct intervention related resources, can be unclear in practice, presenting challenges for costing [6]. The Standards for Reporting Implementation Studies (StaRI) statement provides definitions which were adapted for this study [9]. We have defined implementation costs as encompassing the resources associated with methods or techniques used to enhance the adoption, implementation, or sustainability of a new or under-utilised intervention. This is separate to what may be considered an intervention or technology related cost, which comprise the resources directly associated with the evidence-based practice, programme, policy, process, or guideline recommendation that is being implemented [9].

This paper reports on the development of a pragmatic implementation costing instrument to assist in quantifying the cost of resources used in digital health implementation efforts. The instrument has been iteratively developed across three phases. Phase 1 involved a systematic literature review to identify the nature of implementation costs and the methods used to measure and value these costs, within the context of hospital-based clinical decision support systems initiatives [6]. Phase 2 consisted of qualitative semi-structured interviews that outlined current practices for capturing the costs associated with implementing digital health initiatives in hospital settings [10]. Here we report on the final phase, which aimed to establish consensus regarding core components and design of the implementation costing instrument using a modified electronic-Delphi (e-Delphi) process. In addition to reporting on the methods and findings of the e-Delphi process, this study also aimed to provide an example of intended use of the proposed implementation costing tool using a hypothetical case study.

## Methods

### Study design

We used a modified e-Delphi process to design an implementation costing instrument. The Delphi technique was appropriate to address the study aims as it is a method for establishing consensus among a group of experts [11] and is particularly suitable in areas where there is limited evidence from prior research [12]. While there are various types of Delphi designs [11], the approach is characterised by two or more rounds of questionnaires with controlled feedback, statistical group response, and anonymity via an iterative process until consensus is reached [13].

We used a modified e-Delphi panel design, with the questionnaire administered via an online survey platform. The e-Delphi approach was considered appropriate in this instance as collective subjective assessments were needed, it was difficult to organise group meetings across different time zones and participant schedules, and we wanted to ensure that individual opinions were not masked by more vocal individuals or subgroups [13].

Methods and results are reported in adherence with the reporting standard Conducting and Reporting Delphi Studies (CREDES) [14], which promotes consistency and quality in conducting Delphi studies. Additional file 1 describes how CREDES was observed. Ethical approval was obtained from Metro South Human Research Ethics Committee (HREC/2022/QMS/81677).

### Preliminary work to inform the e-Delphi design

An initial prototype of the implementation costing instrument was developed from the findings of a literature review [6] and qualitative interview study [10]. It was observed in the literature review that there was considerable inconsistency and ambiguity in methods used to cost digital health implementation strategies. However, labour was consistently reported to be the largest implementation-related cost [6]. This was consistent with the views expressed during our prior qualitative interview study with stakeholders which indicated that staff time tracking was a commonly used approach to cost implementation. As a result, the initial prototype included an activity log and a template to capture labour (Additional file 2, page 12). Other implementation-related costs reported in the literature included consumables, durable assets, and physical space. A separate template was included in the prototype to capture these non-labour costs (Additional file 2, page 14).

Our interview study highlighted the need to include a planning template in the initial Cost-IS prototype [10]. Findings from this study indicated that costing implementation was hindered by the perceived ill-defined boundaries of what constitutes implementation strategies, and inconsistencies in terminology used across the disciplines of implementation science, health economics and digital health. The prototype therefore included a planning template to assist users in identifying and classifying implementation costs to allow for appropriate data collection (Additional file 2, page 7).

Our prior work has also highlighted the challenges in consistently identifying and categorising implementation cost data [10]. A key recommendation to arise from our qualitative interview study was that a set of discrete cost categories should be developed prior to the commencement of data collection, in a way that reflects the project/study’s implementation effort. This informed the development of a prototype instrument that used ‘implementation strategies’ as a set of overarching categories to enable mapping resources used to the specific implementation efforts adopted. For each strategy, a set of discrete activities could then be assigned to guide the collection of resource use data. This decision reflected the tool’s grounding in a time-driven activity-based costing approach [2]. A list of common implementation strategies, activities and resources were included in the prototype with explanatory reference text to assist users in completing the planning template (Additional file 2, page 9 and 10). The provided implementation strategies were further categorised across implementation phases based on the widely used Consolidated Framework For Implementation Research (CFIR) (Additional file 2, page 9). The phases aimed to provide clarity on the scope of costing instrument as well as assisting in identifying implementation strategies relevant to the project. The resulting prototype was used as stimulus material in the first round of the e-Delphi (Additional file 2).

### Study participants and recruitment

A purposive sampling approach was used to recruit experts from the academic, government, clinical or health service sectors. An expert was someone who had experience working in the fields of implementation science research, health economic evaluation, and/or digital health. Potential participants were identified through existing collaborative research networks, publicly available hospital and university staff directories, and key academic publications in the fields of implementation science and health economics. Participants from the qualitative interviews in phase two were invited to participate in the e-Delphi panel, as well as new participants. We examined publicly available biographies of potential participants, including academic staff biographies on university websites, to confirm experience in relevant fields. While recruitment primarily occurred within Australia, international participants were also invited. TD emailed an invitation to participate and study information sheet. Consent was collected from those who expressed an interest in participating. An estimated panel size of 10 – 15 participants was preliminarily established from guidelines on conducting Delphi’s in health research [11]. The need for adequate representation across a broad cross-section of participants, as well as pragmatic considerations, determined the final sample size, which consisted of 12 participants.

### Data collection

Participants were sent a link to the questionnaires and the associated stimulus material via email. The prototype implementation costing instrument was provided as the stimulus material. The questionnaires were created in Qualtrics©. The questions were formatted as 10-point numerical rating scale (where 1 = disagree to 10 = agree) that asked the participants to consider a range of elements regarding the implementation costing instrument. Free text spaces were provided for participants to expand on their responses. Cognitive pretesting of the questionnaires using a think aloud method was conducted with two graduate students to establish whether respondents could understand the questions, in a consistent manner, and in a way the researcher intended [15]. Participants were given two-weeks to complete the questionnaire with a reminder email provided after one-week. In subsequent rounds, questions could be changed and/or dropped depending on the findings from the previous round. Round 1 and 2 questionnaires can be found in Additional files 2 and 3, respectively.

The data from the first round was analysed (see ‘Data analysis’ for details) and potential modifications to the implementation costing instrument were discussed within the research team. A refined instrument was subsequently provided as stimulus material in the second round. All updates were made clear to the participants, and consensus on the updates was sought. Also in subsequent round participants were presented with the anonymous results of questions that did not reach consensus in the previous round. The feedback compared each expert’s own answers to the panel which provided the opportunity to reposition their opinion accordingly. The e-Delphi process was terminated when consensus was reached on integral questions, or when three rounds were completed, to prevent sample fatigue [12]. Integral questions were those pertaining specifically to the instrument design and components. Non-integral questions in-directly related to the instrument including its use and users.

### Data analysis

Quantitative data from numerical rating scales was analysed using descriptive statistics generated in Qualtrics©. The percentage of respondents scoring ≥ 7 on a 10-point numerical rating scale was used to determine the percentage agreement score for each item. Consensus was achieved if an item percentage agreement score was ≥ 75% [16]. Percentage agreement scores and frequency bar graphs (generated by Qualtrics©) were presented to the research team for discussion.

The qualitative data from the free-text questions in the rounds was analysed using a thematic content analysis approach. All free-text responses were exported from a Qualtrics© report to a PDF for analysis. Similar suggestions and comments were combined and collapsed into a single suggestion or comment. A summary of all unique suggestions and comments was presented to the research team for discussion.

## Results

### Modified e-Delphi process

Eighteen professionals with experience in implementation science, health economics and/or digital health were invited to participate in the study. Fourteen professionals expressed interest in participating, but two were lost prior to Round 1. The final expert panel of twelve consenting participants contained a sufficient representation of the desired expertise: 50% had expertise in implementation science, 50% had expertise in health economics and 58% had expertise in digital health (Additional file 4: Table 1). Participants included: two implementation scientists, one health economist, three digital health specialists and six with experience across multiple fields (Additional file 4: Fig. 1). Participants worked across a range of healthcare disciplines, clinical areas and settings including nursing, surgery, maternal health, nutrition and dietetics, lung cancer, infectious disease, clinical excellence, and digital health (including telehealth and artificial intelligence). Most participants were female (*n* = 8, 67%), worked in academic contexts (*n* = 11, 92%), and were located in Australia (*n* = 7, 58%) (Additional file 4: Table 2).

In Round 1, consensus was reached on almost all questions, with the exception being a question asking if research activities should be considered as an implementation cost (Additional file 4: Table 3—question 2.3.1: 42% agreement) and two questions regarding the supporting material ‘Appendix C: Common activities and resources to operationalise implementation strategies’ (Additional file 4: Table 3—question 3.5.1: 42% agreement and question 3.5.2: 50% agreement). Percentage agreements from all questions in Round 1 can be found in Additional file 4: Table 3. Feedback and comments from Round 1 resulted in changes to the costing instrument (discussed below). Round 2 of the e-Delphi was used to obtain consensus on the components that did not reach consensus in Round 1 as well as additional questions regarding updates to the instrument made in response to Round 1.

The costing instrument was updated in response to the feedback from Round 1, summarised in Additional file 4: Table 4, and consensus was achieved on these updates in Round 2 (Additional file 4: Table 3—question 4.1.1: 100% agreement; question 4.2.1: 92% agreement; question 4.2.2: 92% agreement). As a result of the consensus on these integral questions, the e-Delphi process was terminated after Round 2. Integral questions related directly to the design and components of the instrument. Non-integral questions were in-directly related to the instrument including its use and users. There were three non-integral items that did not reach consensus in Round 2) that are described below. Percentage agreements from all questions in Round 2 can be seen in Additional file 4: Table 3.

### Areas of non-consensus

#### The nature of research costs

The responses from Round 1 indicated that including research costs as an implementation cost is dependent on the study type and reason. Research costs may include preparing study protocols/ ethics applications, recruiting participants, obtaining consent, managing research data, and dissemination of research findings. Research costs for the purpose of furthering implementation science knowledge may not be relevant when quantifying implementation costs, as these costs would not extend to other institutions or sites considering the implementation of a particular innovation. Conversely, research costs may be relevant to include as an implementation cost when conducting quality improvement studies or when the intervention would otherwise not be implemented without local evidence to support its safety, efficacy, or cost-effectiveness. As a result of this feedback, it was decided to acknowledge research costs as being a potentially relevant implementation cost within the costing instrument, with an explanation that the relevance of research costs is context-specific and should be determined by the user of the instrument. Consensus was achieved on this update to the costing instrument in Round 2 (Additional file 4: Table 3—question 2.1.1: 100% agreement).

#### The user’s prior implementation science knowledge

The initial implementation costing instrument prototype included supporting material designed to provide reference explanations for the user on implementation science concepts including phases, and common implementation strategies, activities, and resources (Additional file 2: Appendix A, page 3; Appendix B, page 9; Appendix C, page 10). The information on implementation phases reached consensus (Additional file 4: Table 3 – question 2.1.2: 75% agreement) but some respondents felt it gave a linear impression of implementation, when such processes are often iterative. Providing examples of common implementation strategies reached consensus (Additional file 4: Table 3—question 3.3.1: 75% agreement) but some respondents suggested inclusion of references to key implementation science articles for those lacking foundational knowledge. The purpose of providing examples of common activities and resources was not clear to participants and (as mentioned above) did not reach consensus (Additional file 4: Table 3—question 3.5.1: 42% agreement and question 3.5.2: 50% agreement). The research team considered that the mixed responses to these instrument supporting materials was likely due to ambiguity in the scope of the instrument.

In response to the feedback from Round 1, the research team decided to refine the purpose and content of the instrument to more clearly align with the intended aim to provide practical, user-friendly templates to assist in the collection of appropriate costing data. It was determined that reference explanations with the intention of educating users on implementation science phases and strategies was beyond the scope of this costing instrument. Hence, the supporting education-related materials were removed from the costing instrument (Additional file 2: Appendix A, page 3; Appendix B, page 9; Appendix C, page 10). This information was replaced with appropriate references to key studies within the implementation science literature to assist users in deepening their understanding as required. These updates were made to the costing instrument in Round 2 and consensus was achieved on both the removal of supporting material (Additional file 4: Table 3—question 4.4.2: 83% agreement) and the refined scope of the instrument (Additional file 4: Table 3—question 3.1.1: 92% agreement).

Through this refinement, the research team recognised that there was an implicit assumption that the user will likely have some prior understanding of implementation science, which we contend is reasonable given the intention to use and cost implementation strategies. In Round 2, we asked the participants if it is appropriate to assume users of the costing instrument will have some level of prior implementation science knowledge; this statement did not reach consensus (Additional file 4: Table 3—question 3.1.3: 67% agreement).

#### Specificity to digital health solutions

The costing instrument was initially framed for application in digital health contexts and there was a suggestion from Round 1 indicating that more digital health specific examples would be helpful. Given the refinements in the overall instrument scope (outlined above), the research team was also prompted to consider making the instrument more generic in nature to allow for potential application beyond digital health contexts. The rationale for this related to the recognition that the costing categories for implementation strategies (as opposed to specific interventions or technologies) used for digital health solutions may be transferrable across settings. Although consensus was not reached on this update to the costing instrument in Round 2 (Additional file 4: Table 3—question 5.1.2: 67% agreement), most participants recognised it was plausible the instrument could be generic. The research team concluded that subsequent piloting would be required to confirm or refute the extent to which the instrument was generalisable beyond digital health.

#### Additional digital formats

In response to the feedback from Round 1, the digital functionality of the costing instrument was improved. An electronic version of the data collection templates was created in Microsoft Excel, including use of ‘drop-down’ options where possible to optimise data quality. The Excel file included two additional summary tables that automatically populated with data entered from the templates. Consensus was achieved on this update to the costing instrument in Round 2 (Additional file 4: Table 3—question 6.1.1: 92% agreement; question 4.1.2: 92% agreement). Participants were satisfied with the Microsoft Excel version and did not indicate interest in any additional digital formats suggested, including REDCap, Microsoft Word, or PDF (Additional file 4: Table 3—question 6.1.3: 33% agreement).

### The final implementation costing instrument

The final costing implementation strategies (Cost-IS) instrument is presented through a worked example below and in Additional file 5. A health system perspective was taken in the worked example. The aim and scope of the instrument is to collect data on the costs associated with implementation strategies for digital health solutions. The instrument comprises of three data collection templates. It can be found online at https://cost-is.github.io/instrument/.

#### Cost-IS template 1: planning

The purpose of Template 1 is to help identify specific data items that need to be collected. This will allow for comprehensive and targeted data collection later in the costing instrument. In Template 1, users document the relevant implementation strategies and then outline which activities are needed to operationalise each of the strategies. Both labour and non-labour resources used to deliver the activities are listed in the final column. Table 1 provides a worked example of Template 1, including four implementation strategies with associated activities and resources.

**Table 1:** Cost-IS template 1 worked example

Strategy	Activities	Resources
Audit and feedback	• Meet with stakeholders to identify outcomes • Retrieve and analyse data on outcomes • Present data to stakeholders	• Project officer • Team leader A • Team leader B • Clinical team A—champion • Clinical team B—champion
Involve existing governing structures	• Meet with executives • Meet with clinical team/s	• Project officer • Executive A • Executive B • Team leader A • Team leader B
Identify and prepare champions	• Engage with stakeholders to identify potential champions • Engage (meetings or emails) with possible champions • Ongoing support for champions	• Project officer • Team leader A • Team leader B • Clinical team A—champion • Clinical team B—champion
Train-the-trainer	• Adapt training with stakeholders • Train the champions to be trainers • Create opportunities for the trainers to train others • Monitor training progress	• Project officer • Team leader A • Team leader B • Clinical team A—champion • Clinical team B—champion • Training material • Training room

#### Cost-IS templates 2A/B: data collection

Templates 2A and 2B are used to collect the data necessary to quantify the implementation costs; 2A collects data on labour resources associated with the implementation strategies, while 2B collects data on non-labour resources. In the worked example of Template 2A (Table 2), all activities associated with the hypothetical implementation were recorded. Each activity instance was given a specific index number because an activity occurred more than once. Similarly, a purpose was recorded for each activity to distinguish it from other similar activities. The implementation strategy related to the respective activity was documented in the same row. Personnel involved in the activity were documented. Each personnel type/role was recorded on a separate row, and roles were distinguished by wage rate or title classification. For each activity, the number of personnel for each role was recorded. Finally, the time spent on the activity for that role was documented. The digital version of this template includes two additional columns which automatically calculate labour costs when the columns presented in Table 2 are completed. In the digital version the entries columns ‘Activity’, ‘Strategy’ and ‘Role’ are restricted by drop down menus containing the items listed in Template 1. Template 1 can be completed iteratively as required by the project.

**Table 2:** Cost-IS template 2A worked example

Index	Activity	Purpose	Strategy	Role	Hourly wage rate ($)	No. of personnel involved	Total person (mins)
1	Meet with executives	present intervention aims and outcomes	Involve existing governing structures	Project officer	85.21	1	30
1	Meet with executives	present intervention aims and outcomes	Involve existing governing structures	Executive A	134.08	1	30
1	Meet with executives	present intervention aims and outcomes	Involve existing governing structures	Executive B	150.48	1	30
2	Meet with clinical team/s	present intervention aims and outcomes	Involve existing governing structures	Project officer	85.21	1	30
2	Meet with clinical team/s	present intervention aims and outcomes	Involve existing governing structures	Team leader A	97.29	1	30
2	Meet with clinical team/s	present intervention aims and outcomes	Involve existing governing structures	Team leader B	115.62	1	30
3	Meet with stakeholders to identify outcomes	meeting to identify what needs to be audited and how to feed it back	Audit and feedback	Project officer	85.21	1	60
3	Meet with stakeholders to identify outcomes	meeting to identify what needs to be audited and how to feed it back	Audit and feedback	Team leader A	97.29	1	60
3	Meet with stakeholders to identify outcomes	meeting to identify what needs to be audited and how to feed it back	Audit and feedback	Team leader B	115.62	1	60
4	Engage with stakeholders to identify potential champions	email asking for champion suggestions	Identify and prepare champions	Project officer	85.21	1	10
4	Engage with stakeholders to identify potential champions	champion suggested via email	Identify and prepare champions	Team leader A	97.29	1	10
4	Engage with stakeholders to identify potential champions	champion suggested via email	Identify and prepare champions	Team leader B	115.62	1	10
5	Engage (meetings or emails) with possible champions	met with clinical team A champion	Identify and prepare champions	Project officer	85.21	1	30
5	Engage (meetings or emails) with possible champions	met with clinical team A champion	Identify and prepare champions	Clinical team A—champion	78.80	1	30
6	Engage (meetings or emails) with possible champions	met with clinical team B champion	Identify and prepare champions	Project officer	85.21	1	30
6	Engage (meetings or emails) with possible champions	met with clinical team B champion	Identify and prepare champions	Clinical team B—champion	56.87	1	30
7	Adapt training with stakeholders	discuss training with stakeholders and adapt to clinical context if needed	Train-the-trainer	Project officer	85.21	1	60
7	Adapt training with stakeholders	discuss training with stakeholders and adapt to clinical context if needed	Train-the-trainer	Team leader A	97.29	1	60
7	Adapt training with stakeholders	discuss training with stakeholders and adapt to clinical context if needed	Train-the-trainer	Team leader B	115.62	1	60
7	Adapt training with stakeholders	discuss training with stakeholders and adapt to clinical context if needed	Train-the-trainer	Clinical team A—champion	78.80	1	60
7	Adapt training with stakeholders	discuss training with stakeholders and adapt to clinical context if needed	Train-the-trainer	Clinical team B—champion	56.87	1	60
8	Adapt training with stakeholders	Incorporate adaptations to training	Train-the-trainer	Project officer	85.21	1	60
9	Train the champions to be trainers	same as activity	Train-the-trainer	Project officer	85.21	1	60
9	Train the champions to be trainers	same as activity	Train-the-trainer	Clinical team A—champion	78.80	1	60
9	Train the champions to be trainers	same as activity	Train-the-trainer	Clinical team B—champion	56.87	1	60
10	Create opportunities for the trainers to train others	book meeting room for monthly training sessions for champions to train	Train-the-trainer	Project officer	85.21	1	15
11	Ongoing support for champions	check in with champions	Identify and prepare champions	Project officer	85.21	1	30
12	Monitor training progress	request current training numbers	Train-the-trainer	Project officer	85.21	1	10
13	Retrieve and analyse data on outcomes	same as activity	Audit and feedback	Project officer	85.21	1	30
14	Ongoing support for champions	check in with champions	Identify and prepare champions	Project officer	85.21	1	30
15	Monitor training progress	request current training numbers	Train-the-trainer	Project officer	85.21	1	10
16	Retrieve and analyse data on outcomes	same as activity	Audit and feedback	Project officer	85.21	1	30
17	Present data to stakeholders	ensure stakeholders are happy with progress, and address any issues	Audit and feedback	Project officer	85.21	1	30
17	Present data to stakeholders	ensure stakeholders are happy with progress, and address any issues	Audit and feedback	Clinical team A—champion	78.80	1	30
17	Present data to stakeholders	ensure stakeholders are happy with progress, and address any issues	Audit and feedback	Clinical team B—champion	56.87	1	30
17	Present data to stakeholders	ensure stakeholders are happy with progress, and address any issues	Audit and feedback	Team leader A	97.29	1	30
17	Present data to stakeholders	ensure stakeholders are happy with progress, and address any issues	Audit and feedback	Team leader B	115.62	1	30

#### Summary table examples

**Fig. 1:**
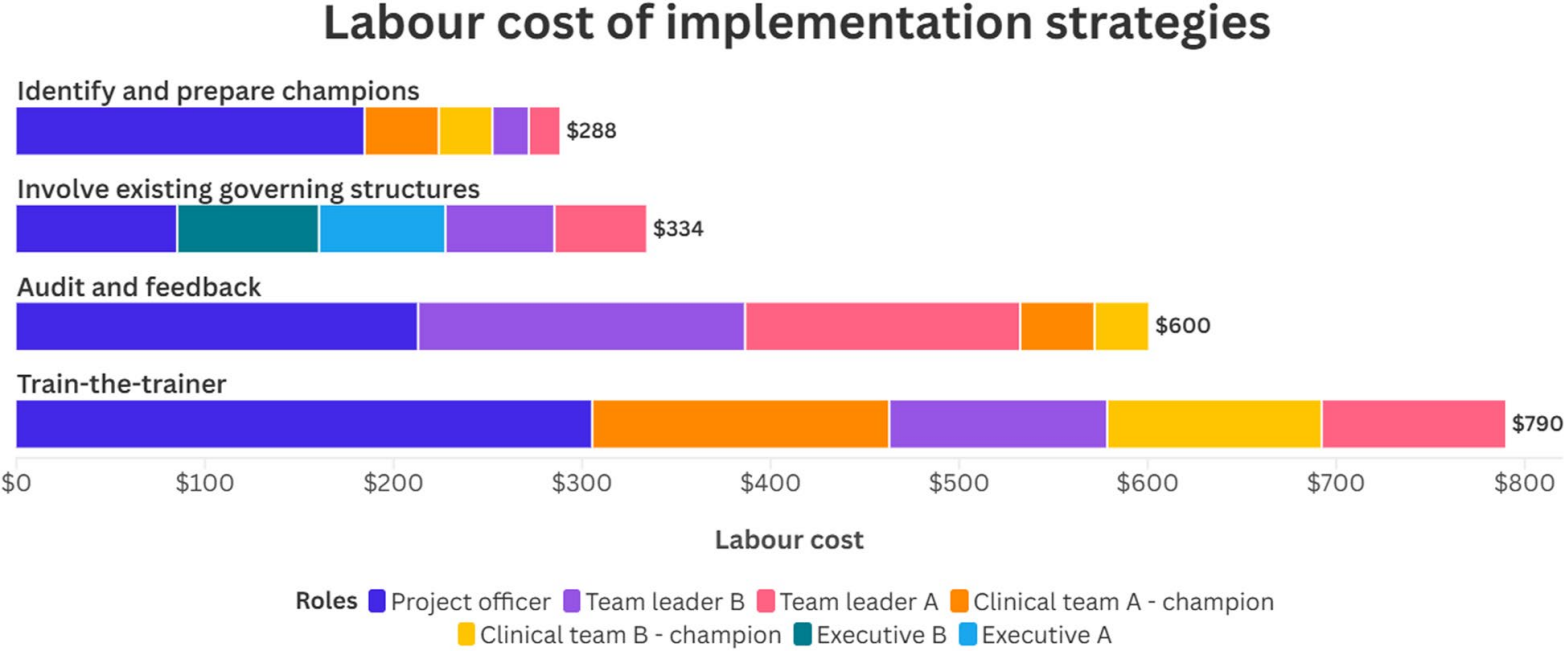
Cost-IS summary figure worked example (role and strategy)

Summary tables can be readily created from the data in the completed templates in a meaningful way as determined by the analyst. The templates were designed to collect data at varying levels of detail because of the wide range and adaptable nature of implementation projects. Table 3 and Fig. 1 demonstrates how implementation costs from the worked example can be summarised by both role and implementation strategy.

**Table 3:** Cost-IS summary table worked example (role and strategy)

Personnel	Implementation strategies				Labour totals ($)
	Train-the-trainer ($)	Audit and feedback ($)	Involve existing governing structures ($)	Identify and prepare champions ($)	
Project officer	305.34	213.03	85.21	184.62	**788.19**
Team leader B	115.62	173.43	57.81	19.27	**366.13**
Team leader A	97.29	145.94	48.65	16.22	**308.09**
Clinical team A—champion	157.61	39.40	-	39.40	**236.41**
Clinical team B—champion	113.75	28.44	-	28.44	**170.62**
Executive B	-	-	75.24	-	**75.24**
Executive A	-	-	67.04	-	**67.04**
**Total**	**789.60**	**600.23**	**333.94**	**287.94**	**2,011.72**

## Discussion

We have presented a new instrument to cost implementation strategies for digital health solutions, Cost-IS, which was developed through the refinement in the modified e-Delphi process of an initial prototype based on a prior literature review [6] and interviews [10] with invested constituents. Cost-IS was developed to utilise existing project documentation to estimate cost. Cost-IS provides a pragmatic yet structured instrument for collecting costs of implementation strategies, which is an important contribution to emerging research efforts in this field [6].

Despite recent calls for a greater focus on cost within implementation evaluations [3, 17, 18], the Implementation Outcome Repository does not yet contain any validated implementation costing instruments [19]. The Repository’s criteria likely limited its ability to include costing instruments noting that previous reviews have identified eight implementation costing instruments [19]. Yet, only one of the eight identified implementation costing instruments was focused on measuring implementation costs that fit the definition in our study [20]. In the previous review, cost was defined as the financial impact of an implementation effort which included costs associated with the intervention, the implementation strategies used, and the location of service delivery. Cost-IS is focused on costing implementation strategies to support appropriate resourcing of implementation efforts and to address the knowledge gap in implementation science research [10].

The cost of implementing new strategies (COINS) is one of few approaches reported in the literature to cost implementation strategies. COINS is a cost-mapping tool developed as an adjunct to an eight-stage framework that measures the implementation process and milestones, the Stages of Implementation of Completion (SIC) [21, 22]. SIC is a comprehensive and structured framework, with a high level of detail that may not be required in some implementation projects [23]. In contrast, Cost-IS is designed as a flexible instrument where the level of detail can be tailored to meet the requirements of a wide range of implementation projects. SIC and COINS were initially created for the foster care setting [24], and a universal version has subsequently been developed for intended use in justice system, schools, and public health settings [25]. Cost-IS was developed in the setting of digital health and intended for use in digital health implementation projects, but may also have utility beyond this domain; however examining this was beyond the scope of the present study.

Another approach to cost implementation strategies was recently developed by Cidav and colleagues [2]. The pragmatic approach combines an established business accounting technique (time-driven activity-based costing- TDABC) with an implementation science framework (the Proctor framework) to cost implementation strategies [2]. TDABC is similar to staff time tracking which Cost-IS was developed from because it was a common method used for costing implementation in practice [10]. As a result, Cost-IS shares some similarities with the TDABC pragmatic method. The main difference is that the TDABC costing matrix reports temporality and frequency whereas Cost-IS does not. Cost-IS aims to utilise already collected data, including field notes or logs of implementation progress, to reduce the burden of data collection and enable implementation costing [10]. As a result, Cost-IS allows for the entry of single occurrence implementation activities with the inclusion of a ‘purpose’ column to track progress. TDABC’s pragmatic method does not have a specific template for non-labour costs (unlike Cost-IS Template 2B) but the methods do acknowledge and prompt users to consider these cost. Both methods allow for comparison of how specific implementation components influence the overall cost.

The Cost-IS instrument developed from this study has potential to address a key knowledge gap in implementation science by providing practical implementation costing [10]. It is our intention that this instrument will improve the robustness of implementation strategy costings, which may in turn lead to more appropriate resource allocation for implementation strategies in future projects, in turn supporting overall project outcomes. Cost-IS enables granular analysis of the costs associated with various strategies, phases and roles, supporting further reflection and analysis around the relative value for money associated with different components within an overarching implementation effort. Different cost perspectives can be taken when using the tool, accommodating for various study designs. Cost-IS aims to advance implementation science methods by providing structured support for data collection and reporting of implementation costs.

The expert panel highlighted potential limitations to use of Cost-IS during the e-Delphi process including the user’s level of implementation science knowledge, the specificity of the tool to digital health and accessibility via digital formats (discussed in the results section). The expert panel also expressed concerns regarding the potential for recall bias and imprecision from a single occurrence of data collection. These are important considerations, with the user needing to determine what is appropriate for their project.

The findings in this study are limited by the data collected during the e-Delphi process. One consideration was the sampling approach. It was important to sample across the different fields of implementation science, health economics, and digital health because the resulting instrument needed to be intrinsically transdisciplinary to reflect its intended use in practice. Heterogenous panels can increase the validity of the results, particularly when consensus is reached [26]. While we achieved sufficient heterogeneity in field domain, other contexts were more homogenous. For example in our study, most participants were presently working in academic contexts and based in Australia, therefore the extent to which Cost-IS generalises beyond academia and dissimilar contexts internationally is uncertain.

## Conclusion

Cost-IS is an instrument for costing implementation strategies for digital health solutions, developed using a modified e-Delphi process with twelve experts in implementation science research, health economic evaluations and/or digital health. The instrument provides a pragmatic and flexible approach that can be tailored to meet the needs of various projects. Further piloting of Cost-IS is required to validate its feasibility and generalisability.

## Supplementary Information

Additional file 1. CREDES.

Additional file 2. Questionnaire and stimulus material for Round 1 of e-Delphi.

Additional file 3. Questionnaire and stimulus material for Round 2 of e-Delphi.

Additional file 4. Participant characteristics and e-Delphi results.

Additional file 5. The costing implementation strategies (Cost-IS) instrument- a worked example.

## Data Availability

The authors declare that the data supporting findings of this study are available within the paper.

## Abbreviations

COINS: Cost of implementing new strategies
Cost-IS: Costing Implementation Strategies instrument
CREDES: Conducting and REporting DElphi Studies
e-Delphi: Electronic-Delphi
HREC: Human Research Ethics Committee
SIC: Stages of Implementation of Completion
StaRI: The Standards for Reporting Implementation Studies
