# Supplementary material for: Development of an instrument (Cost-IS) to estimate costs of implementation strategies for digital health solutions: a modified e-Delphi study
Source: Implement Sci. 2025 Mar 7;20:13. doi: 10.1186/s13012-025-01423-w (PMC11889902; doi:10.1186/s13012-025-01423-w)

Additional File 2: Questionnaire and stimulus material for Round 1 of e-Delphi

**Questionnaire**

Start of Block: Introduction

Q1.1

Introduction
 Thank you for your participation in this e-Delphi panel!

 The **purpose of this study** is to capture the opinions of experts in implementation science, health economics, and digital health in relation to developing an implementation costing instrument for digital health initiatives. Digital health technologies can include mobile health applications, electronic health (e-health) records, telehealth/medicine, wearable devices, and artificial intelligence, among others.

 The e-Delphi panel will consist of up to 3 rounds, with a questionnaire to complete in each round. All rounds will be conducted electronically via Qualtrics. The anonymous results of the previous round will be supplied to each participant along with their answers to allow the participant to reflect on their opinion prior to answering the following questionnaire.

 Please familiarise yourself with the stimulus material prior to completing the questionnaire. If completing this questionnaire on a phone you may want to have a printed copy of the stimulus material.

 Participation in each round will take **30 – 45 minutes**. Participants will have **2-weeks to complete** each questionnaire round, with a reminder email provided at 1-week.

 For more details on this study please refer to the Participant Information Sheet. 


 Please enter your first and last name. This information allows personalised results to be returned to the correct participant. All answers will be anonymous to the Delphi group participants, but not to the research team.

________________________________________________________________

Q1.2

Please complete the following consent form to continue to the questionnaire.


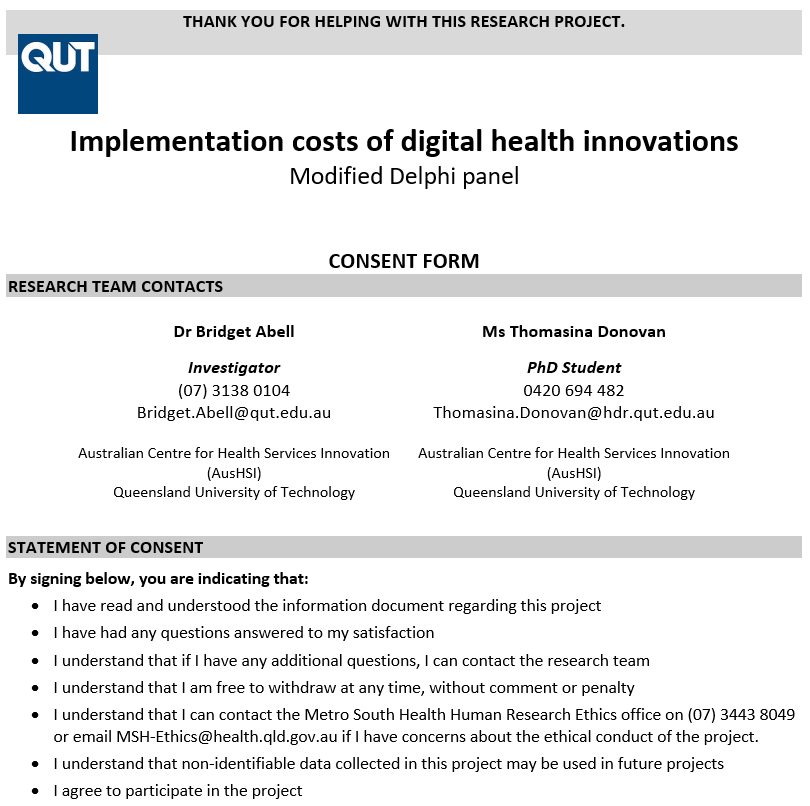


- Yes (1)
- No (2)

Skip To: End of Survey If Please complete the following consent form to continue to the questionnaire. != Yes

End of Block: Introduction

Start of Block: Definition

Q2.1

Questions 2.1 - 2.4 relate to the following information.

Background information

We aim to develop an implementation costing instrument because there are none that can record costs associated with the implementation of digital health initiatives. We want to define the scope of the costing instrument so users know what costs can be captured in instrument. We propose the scope of the costing instrument will include costs associated with the implementation process across following phases: planning, engaging, executing, and reflecting and evaluating. This scope is based on the Consolidated Framework For Implementation Research (CFIR) description of the implementation process. Activities conducted in each phase can be accomplished formally or informally, in any order, and are often conducted in a spiral, stop-and-start, or an incremental approach. Additionally, each phase can be revisited, expanded, refined, and re-evaluated throughout the implementation process. The following Table (Appendix A) summarises the phases, please refer to the linked paper for a detailed explanation.

Appendix A: Proposed scope for the implementation costing instrument


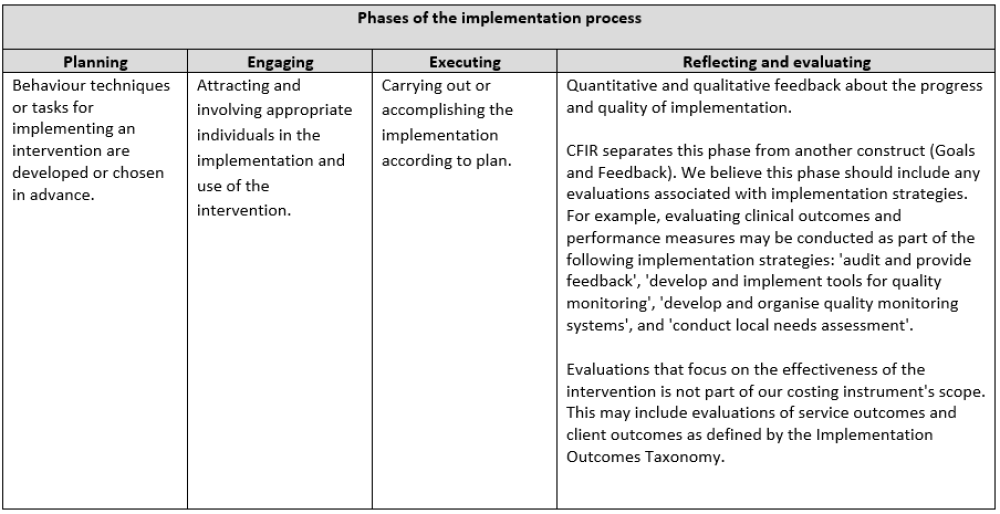


The following questions relate to the proposed costing instrument's scope in Appendix A. Please indicate the extent to which you agree/disagree with the following statements.

|  | Disagree | Neutral | Agree |
| --- | --- | --- | --- |

|  | 1 | 2 | 3 | 4 | 5 | 6 | 7 | 8 | 9 | 10 |
| --- | --- | --- | --- | --- | --- | --- | --- | --- | --- | --- |

| It is important to define the implementation process (planning, engaging, executing, reflecting and evaluating) in the instrument's scope. () | 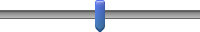 |
| --- | --- |
| The proposed scope (planning, engaging, executing, reflecting and evaluating) adequately captures implementation costs. () | 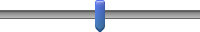 |

Q2.2

Please expand on your answers above and explain why you agree/disagree in the space supplied below. The following prompts may help:

- are the phases (planning, engaging, executing, reflecting and evaluating) appropriate?
- should anything be removed/ added from the scope?

________________________________________________________________

________________________________________________________________

________________________________________________________________

________________________________________________________________

________________________________________________________________

Q2.3

Please indicate the extent to which you agree/disagree with the following statement.

|  | Disagree | Neutral | Agree |
| --- | --- | --- | --- |

|  | 1 | 2 | 3 | 4 | 5 | 6 | 7 | 8 | 9 | 10 |
| --- | --- | --- | --- | --- | --- | --- | --- | --- | --- | --- |

| I believe research activities are an implementation cost, in an implementation study. (Research activities can include preparing study protocols/ ethics applications, recruiting participants, obtaining consent, managing research data, and dissemination of research findings.) () | 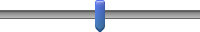 |
| --- | --- |

Q2.4

Please expand on your answer above and explain why you agree/disagree in the space supplied below.

________________________________________________________________

________________________________________________________________

________________________________________________________________

________________________________________________________________

________________________________________________________________

End of Block: Definition

Start of Block: Identification

Q3.1

The first step in the proposed costing instrument will be to identify implementation costs with the aid of Table 1, Appendix B, and Appendix C.

Figure 1: Flowchart of how to use the implementation costing instrument.


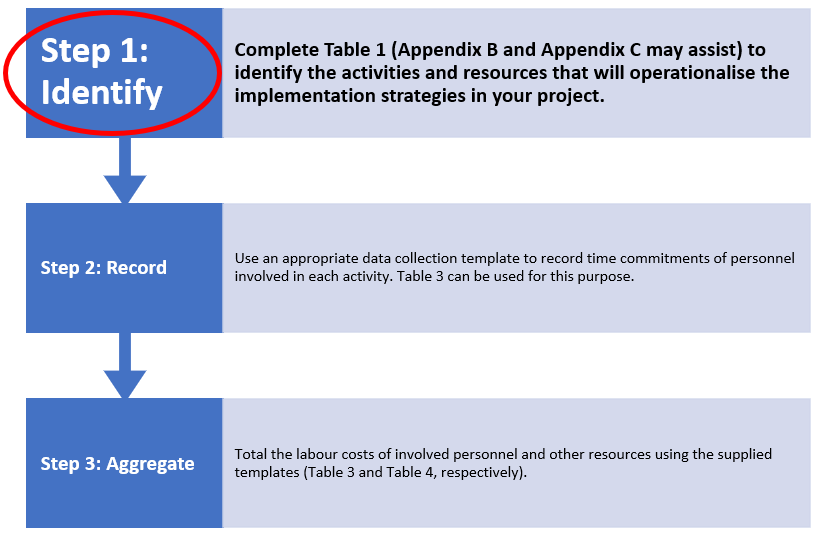


The purpose of **Table 1** is to identify the activities and resources required to operationalise implementation strategies. This will allow for comprehensive and targeted data collection later in the costing instrument where users will assign monetary values to these activities and resources.

Table 1: Identification of activities and resources used to operationalise implementation strategies in a project.


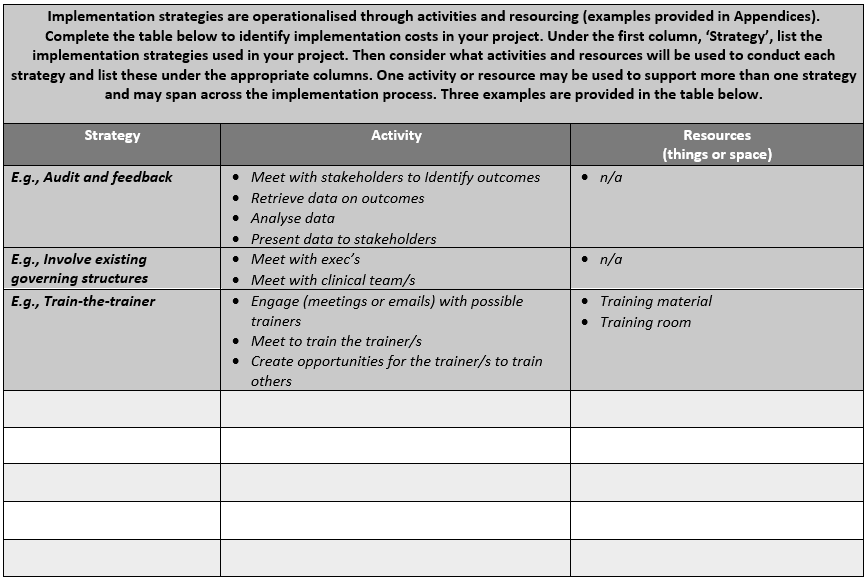


The following questions relate to Table 1 and its use in helping to identify costs associated with the implementation of digital health initiatives. Please indicate the extent to which you agree/disagree with each of the statements below.

|  | Disagree | Neutral | Agree |
| --- | --- | --- | --- |

|  | 1 | 2 | 3 | 4 | 5 | 6 | 7 | 8 | 9 | 10 |
| --- | --- | --- | --- | --- | --- | --- | --- | --- | --- | --- |

| Individuals other than implementation scientists could complete Table 1. () | 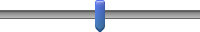 |
| --- | --- |
| Table 1 is suitably flexible for use across a range of initiatives. () | 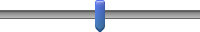 |
| Table 1 is practical (i.e., functional in design) if supplied in printable and electronic (e.g., word processing, spreadsheet) formats. () | 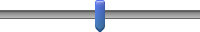 |
| Table 1 is a useful aid for identifying implementation costs (i.e., has value). () | 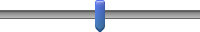 |

Q3.2

Please expand on your answers above and explain why you agree/disagree in the space supplied below. The following prompts may help:

- does Table 1 contain too much jargon?
- what type of digital health implementations may not be appropriate for Table 1?
- how could Table 1 be more practical?
- how could Table 1 add more value to the implementation of digital health?

________________________________________________________________

________________________________________________________________

________________________________________________________________

________________________________________________________________

________________________________________________________________

Q3.3

**Appendix B** includes commonly used implementation strategies in digital health implementation projects. It was informed from earlier studies (interviews and a systematic review). The purpose of Appendix B is to help users complete Table 1.

Appendix B: Common implementation strategies in digital health implementation.


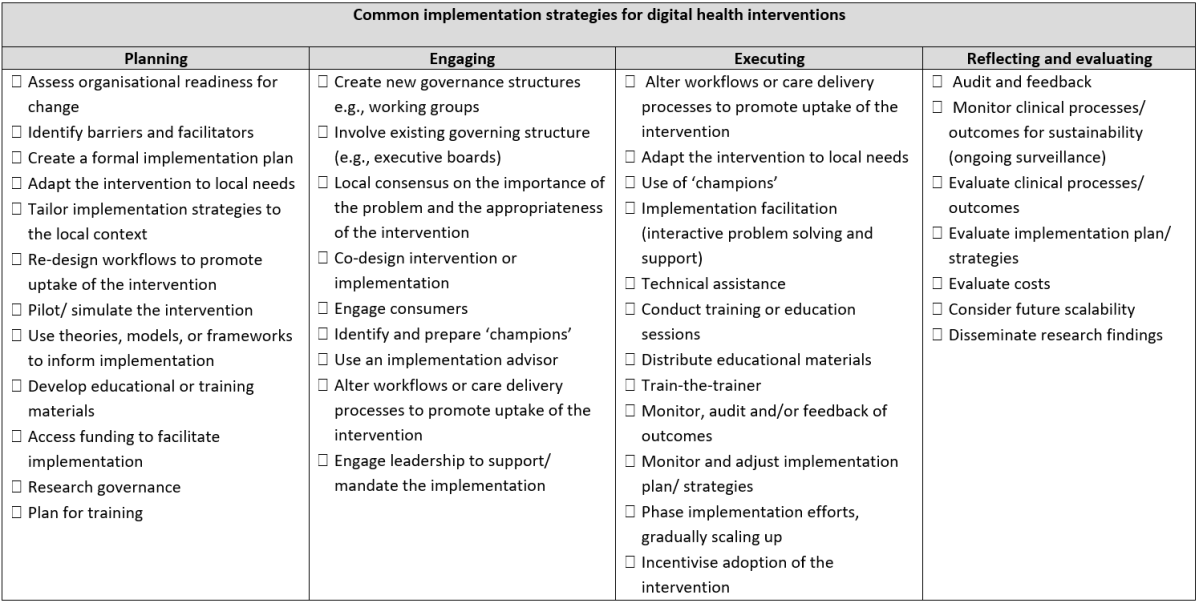


The following questions relate to Appendix B and its use in helping to identify costs associated with the implementation of digital health initiatives. Please indicate the extent to which you agree/disagree with each of the statements below.

|  | Disagree | Neutral | Agree |
| --- | --- | --- | --- |

|  | 1 | 2 | 3 | 4 | 5 | 6 | 7 | 8 | 9 | 10 |
| --- | --- | --- | --- | --- | --- | --- | --- | --- | --- | --- |

| The listed implementation strategies are comprehensive (i.e., key strategies are present). () | 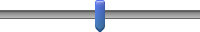 |
| --- | --- |
| Categorising the implementation strategies by implementation phase is useful (i.e., makes it easier to use). () | 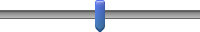 |

Q3.4

Please expand on your answers above and explain why you agree/disagree in the space supplied below. The following prompts may help:

- are any strategies missing or irrelevant?
- is there a better way to categorise the strategies?
- do the strategies need to be categorised?
- does Appendix B have too much jargon?

________________________________________________________________

________________________________________________________________

________________________________________________________________

________________________________________________________________

________________________________________________________________

Q3.5

**Appendix C** suggests common activities and resources needed to operationalise implementation strategies. The purpose of Appendix C is to help users complete Table 1.

Appendix C: Common activities and resources used to operationalise implementation strategies.

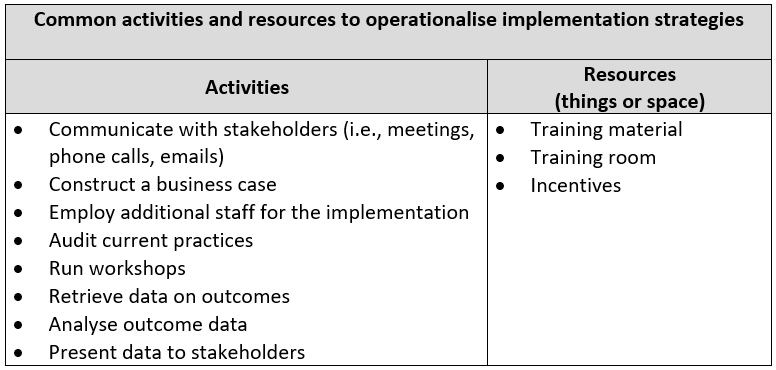


The following question relates to Appendix C and its use in helping to identify costs associated with the implementation of digital health initiatives. Please indicate the extent to which you agree/disagree with the statement below.

|  | Disagree | Neutral | Agree |
| --- | --- | --- | --- |

|  | 1 | 2 | 3 | 4 | 5 | 6 | 7 | 8 | 9 | 10 |
| --- | --- | --- | --- | --- | --- | --- | --- | --- | --- | --- |

| The listed activities and resources are comprehensive (i.e., key ones are present). () | 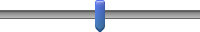 |
| --- | --- |
| Appendix C is a valuable inclusion in the instrument. () | 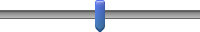 |

Q3.6

Please expand on your answers above and explain why you agree/disagree in the space supplied below. The following prompts may help:

- are any key activities or resources missing or irrelevant?
- are the activities and resources clear?
- does it need to be expanded?

________________________________________________________________

________________________________________________________________

________________________________________________________________

________________________________________________________________

________________________________________________________________

End of Block: Identification

Start of Block: Data collection

Q4.1

The second step in the proposed costing instrument is to collect data on implementation costs with the aid of Table 2 (below).

Figure 1: Flowchart of how to use the implementation costing instrument.


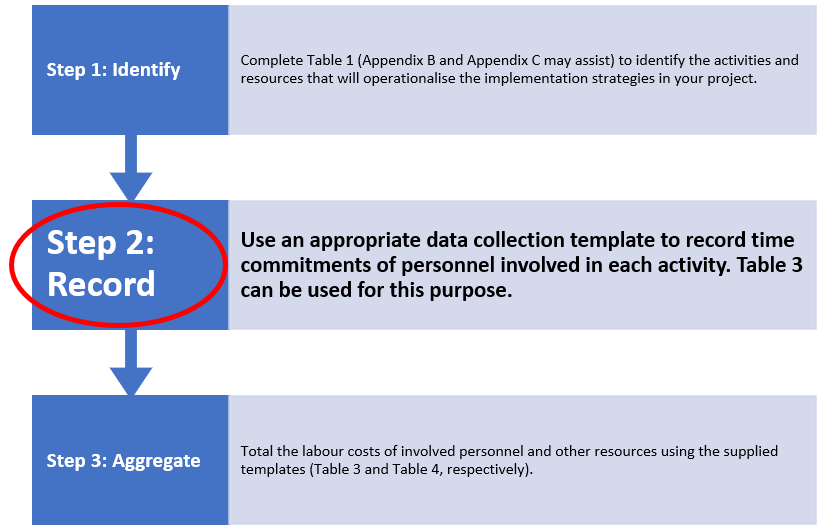


**Table 2** is an activity log template. Its purpose is to aid in the collection of appropriate data to adequately capture costs associated with implementing digital health initiatives.

Table 2: Activity log template

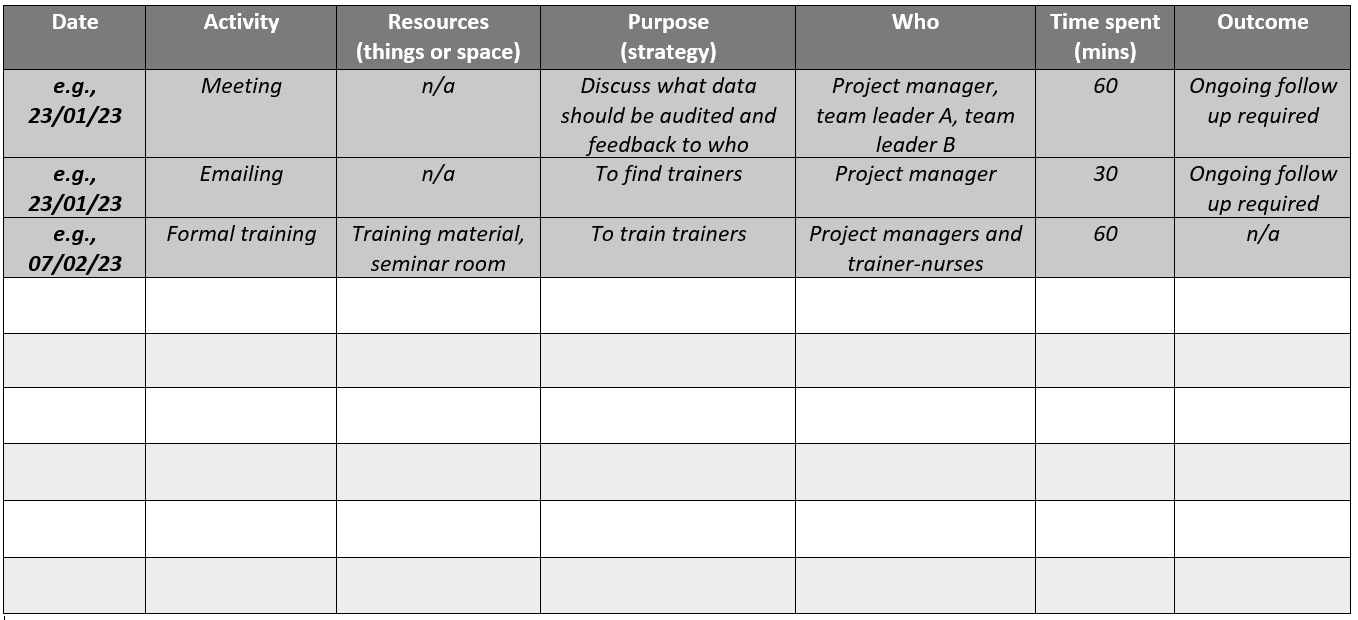


The following questions relate to Table 2 and its assistance in the collection of data on costs associated with the implementation of digital health initiatives. Please indicate the extent to which you agree/disagree with each of the statements below.

|  | Disagree | Neutral | Agree |
| --- | --- | --- | --- |

|  | 1 | 2 | 3 | 4 | 5 | 6 | 7 | 8 | 9 | 10 |
| --- | --- | --- | --- | --- | --- | --- | --- | --- | --- | --- |

| Table 2 can comprehensively collect data on implementation costs (i.e., key data can be captured). () | 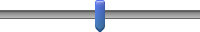 |
| --- | --- |
| Table 2 is a useful aid (i.e., has value) to collect data on implementation costs. () | 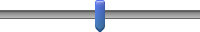 |

Q4.2

Please expand on your answers above and explain why you agree/disagree in the space supplied below. The following prompts may help:

- how could the Table 2 be improved?
- is information missing or irrelevant in Table 2?
- is Table 2 appropriate, user friendly, pragmatic, feasible?

________________________________________________________________

________________________________________________________________

________________________________________________________________

________________________________________________________________

________________________________________________________________

End of Block: Data collection

Start of Block: Data aggregation

Q5.1

The third step in the proposed costing instrument is to aggregate the collected data on implementation costs with the aid of Table 3 and Table 4 (below).

Figure 1: Flowchart of how to use the implementation costing instrument.


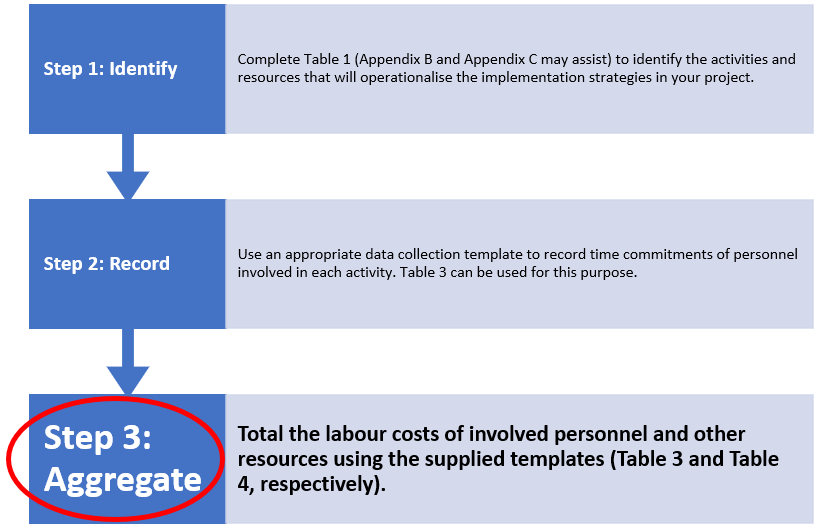


The purpose of **Table 3** is to aggregate labour costs associated with implementing digital health initiatives.

Table 3: Aggregate labour costs template
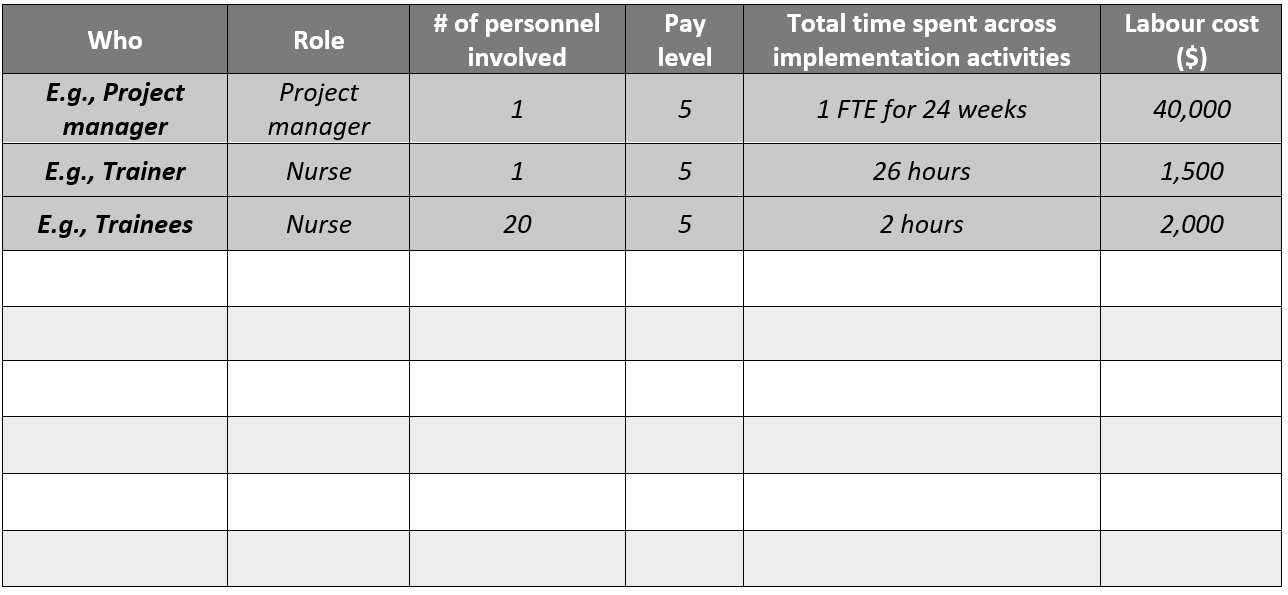


The following questions relate to Table 3 and its assistance in the aggregation of data on costs associated with the implementation of digital health initiatives. Please indicate the extent to which you agree/disagree with each of the statements below.

|  | Disagree | Neutral | Agree |
| --- | --- | --- | --- |

|  | 1 | 2 | 3 | 4 | 5 | 6 | 7 | 8 | 9 | 10 |
| --- | --- | --- | --- | --- | --- | --- | --- | --- | --- | --- |

| Table 3 can comprehensively aggregate data on implementation labour costs (i.e., key costs can be collated). () | 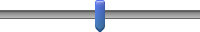 |
| --- | --- |
| Table 3 is a useful aid (i.e., has value) to aggregate data on implementation labour costs. () | 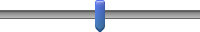 |

Q5.2

Please expand on your answers above and explain why you agree/disagree in the space supplied below. The following prompts may help:

- how could Table 3 be improved?
- is information missing or irrelevant in Table 3?
- is Table 3 appropriate, user friendly, pragmatic, feasible?

________________________________________________________________

________________________________________________________________

________________________________________________________________

________________________________________________________________

________________________________________________________________

Q5.3

The purpose of **Table 4** is to aggregate the costs associated with the resources (things and space) needed for implementing digital health initiatives.

Table 4: Aggregate resource (things and space) costs template


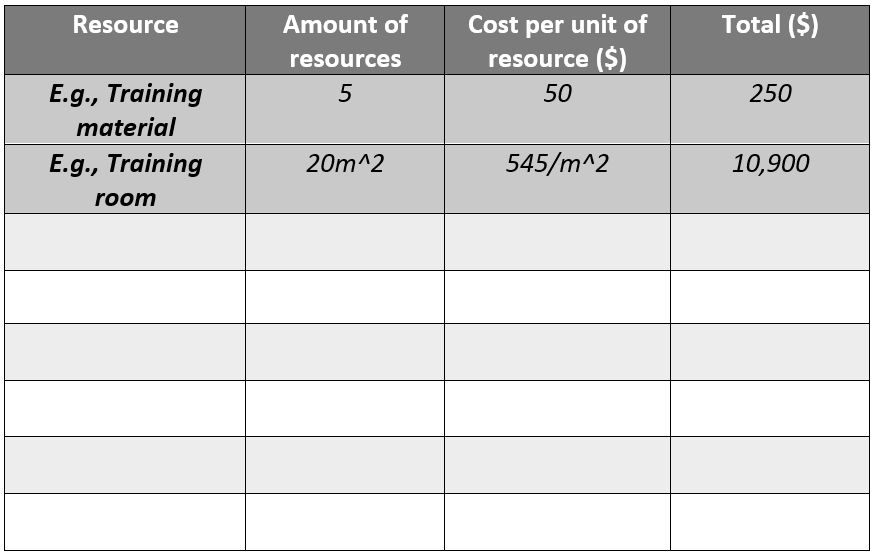


The following questions relate to Table 4 and its assistance in the aggregation of data on costs associated with the implementation of digital health initiatives. Please indicate the extent to which you agree/disagree with each of the statements below.

|  | Disagree | Neutral | Agree |
| --- | --- | --- | --- |

|  | 1 | 2 | 3 | 4 | 5 | 6 | 7 | 8 | 9 | 10 |
| --- | --- | --- | --- | --- | --- | --- | --- | --- | --- | --- |

| Table 4 can comprehensively aggregate data on implementation resource costs (i.e., key costs can be collated). () | 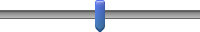 |
| --- | --- |
| Table 4 is a useful aid (i.e., has value) tp aggregate data on implementation resource costs. () | 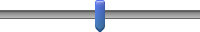 |

Q5.4

Please expand on your answers above and explain why you agree/disagree in the space supplied below. The following prompts may help:

- how could Table 4 be improved?
- is information missing or irrelevant in Table 4?
- is Table 4 appropriate, user friendly, pragmatic, feasible?

________________________________________________________________

________________________________________________________________

________________________________________________________________

________________________________________________________________

________________________________________________________________

End of Block: Data aggregation

Start of Block: Conclusion

Q6.1

Please provide any other comments in the space supplied below. Additional comments may be about:

- our proposed implementation costing instrument
- implementation costs
- costing instruments in general
- considerations for digital health initiatives

________________________________________________________________

________________________________________________________________

________________________________________________________________

________________________________________________________________

________________________________________________________________

End of Block: Conclusion

**Stimulus Material**


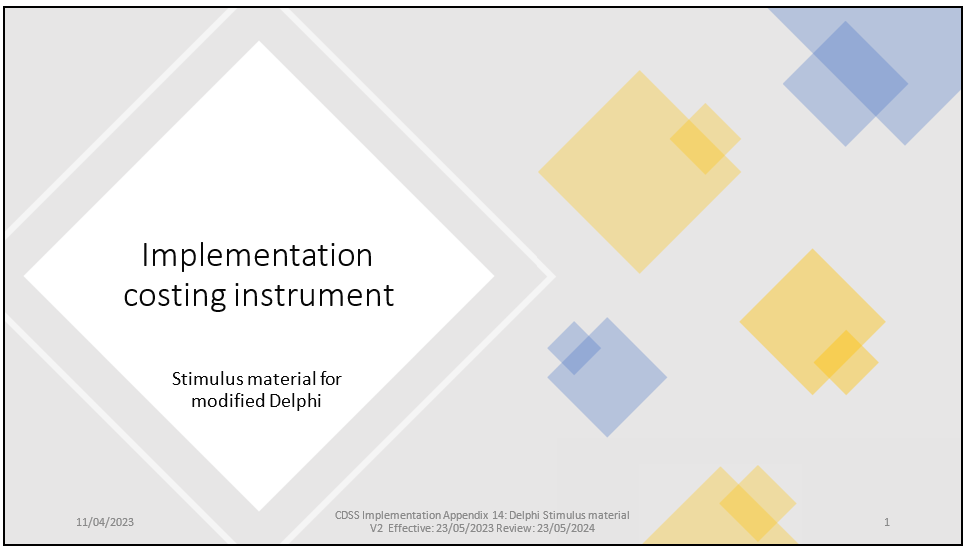


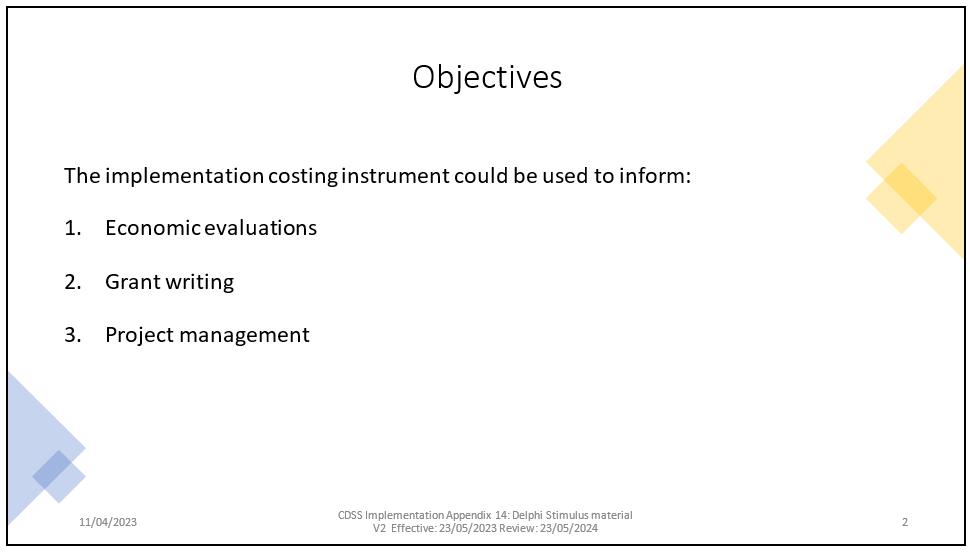


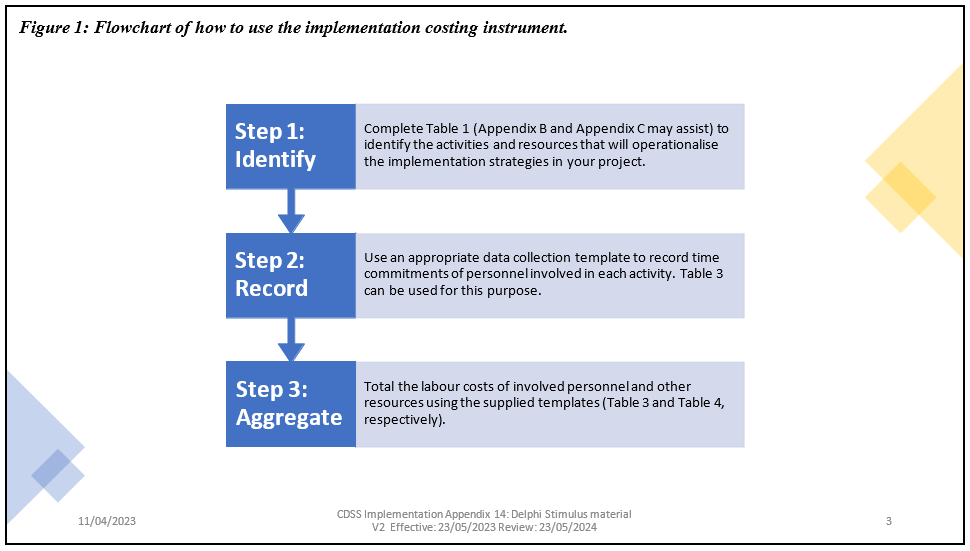


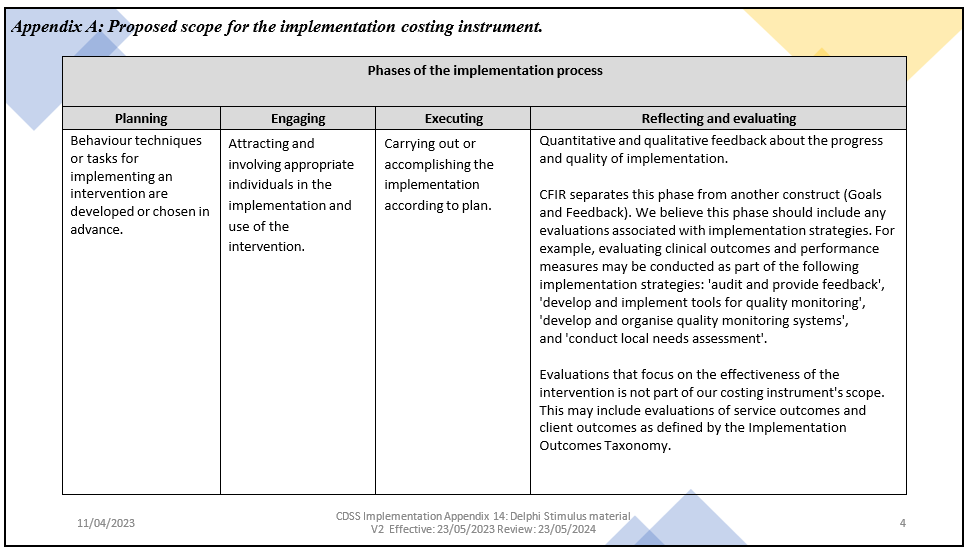


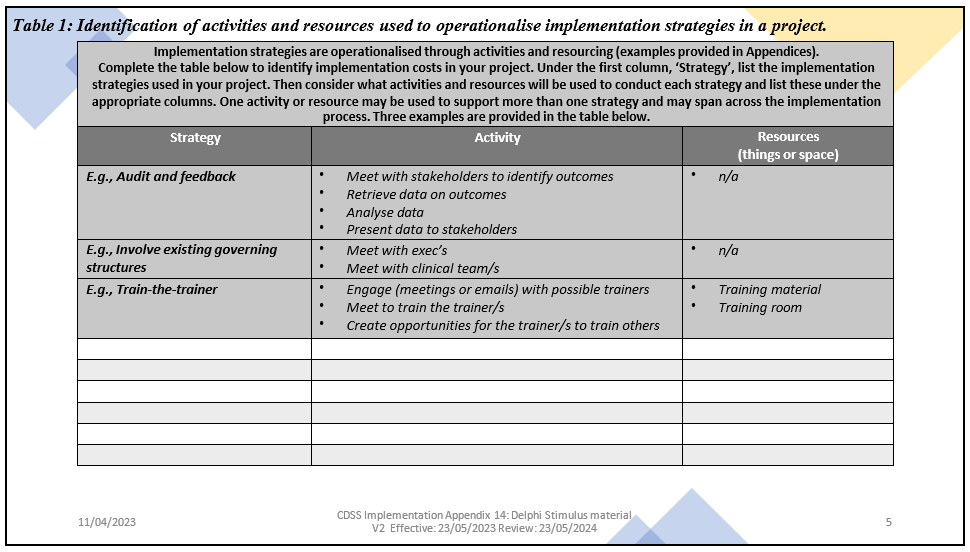


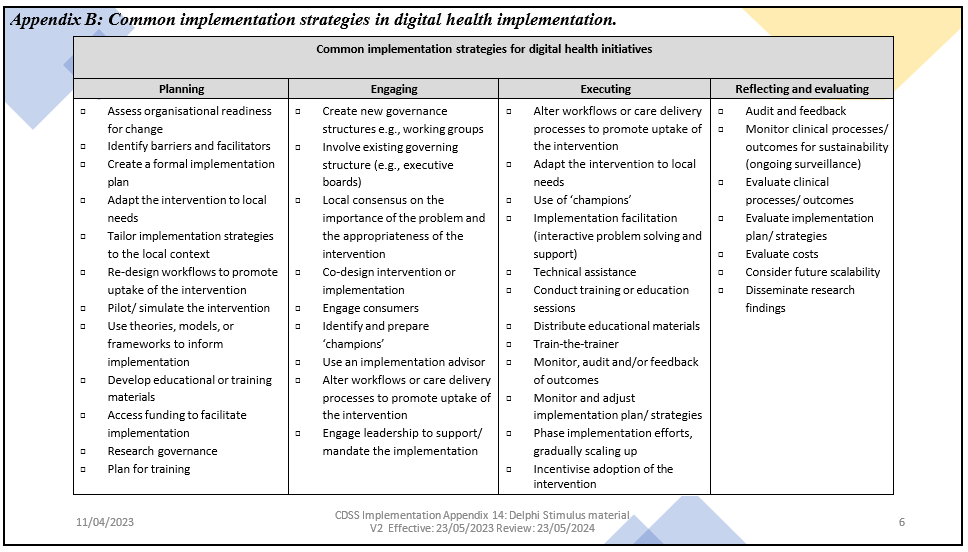


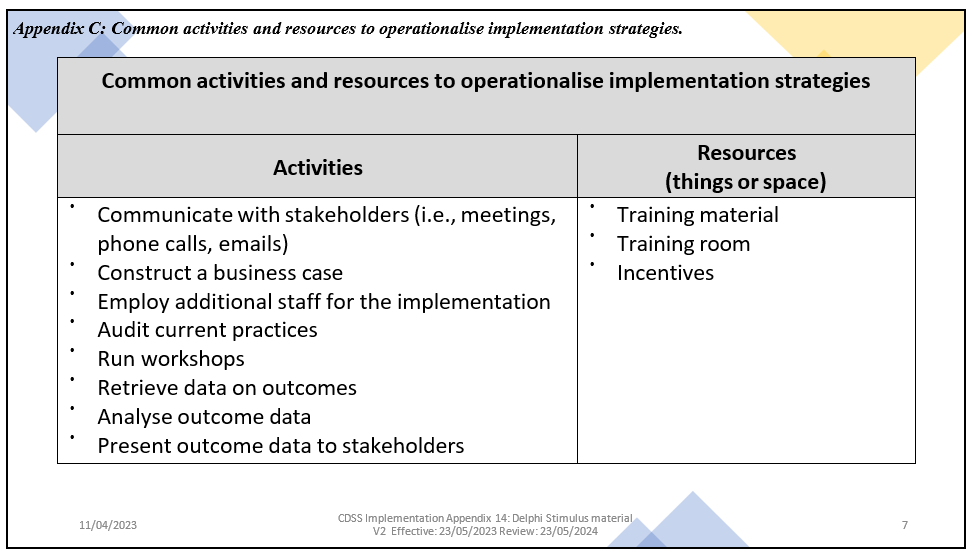


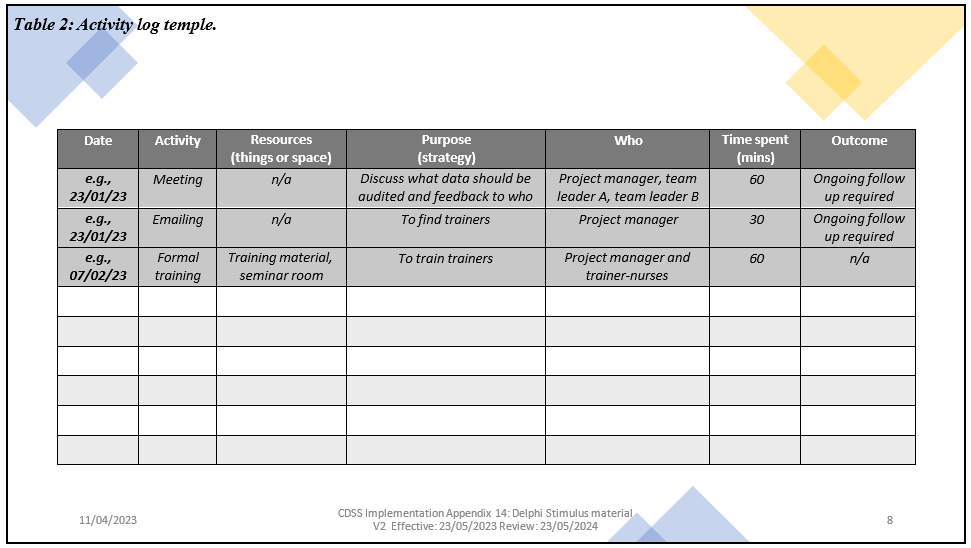


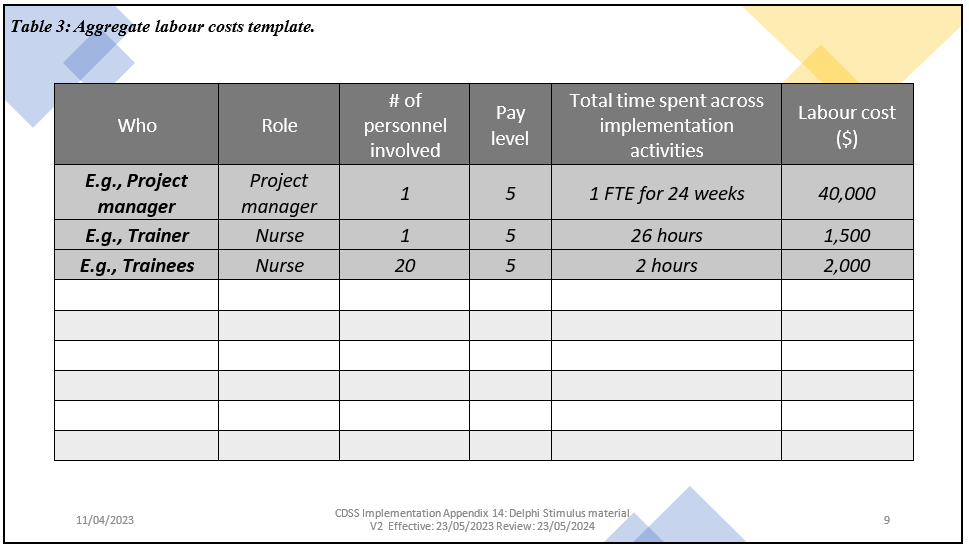


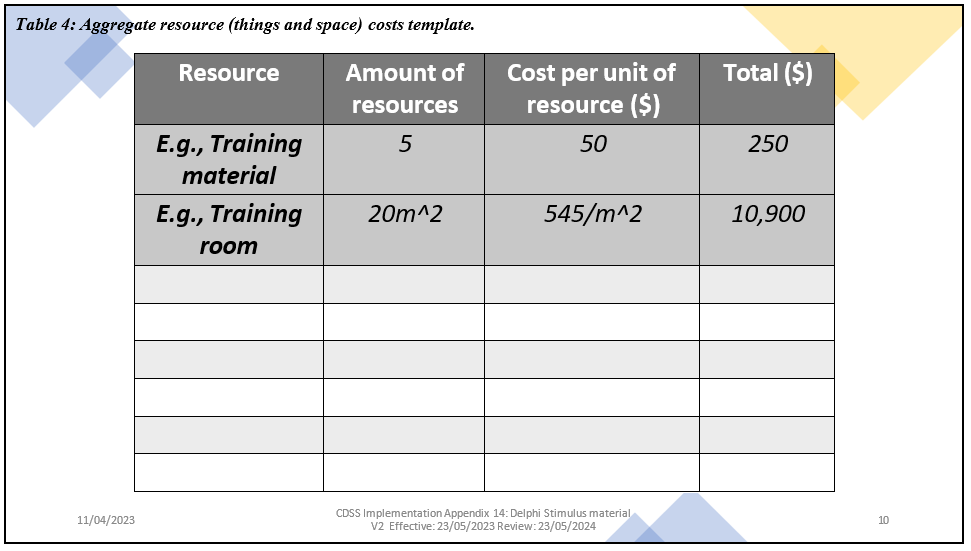

Supplement: Supplementary file 2 — Additional file 2. Questionnaire and stimulus material for Round 1 of e-Delphi. [file 13012_2025_1423_MOESM2_ESM.docx]
