# Supplementary material for: Development of an instrument (Cost-IS) to estimate costs of implementation strategies for digital health solutions: a modified e-Delphi study
Source: Implement Sci. 2025 Mar 7;20:13. doi: 10.1186/s13012-025-01423-w (PMC11889902; doi:10.1186/s13012-025-01423-w)

Additional File 3: Questionnaire and stimulus material for Round 2 of e-Delphi

**Questionnaire**

Start of Block: Introduction

Q1.1 Introduction

Thank you for your continued participation in this e-Delphi panel!

 Round 1 findings- Overview
Consensus was reached on all questions except for:

- the question asking if research activities should be considered as an implementation cost
- two questions regarding the supporting material Appendix C: Common activities and resources to operationalise implementation strategies.

Despite this high level of consensus, the insightful feedback from Round 1 resulted in several improvements being made to the costing instrument. Therefore, in this round of the Delphi, questions will largely focus on the changes made to the instrument. 

It’s important to read through the information provided to understand how and why the instrument has changed before answering the questions.  

Please enter your first and last name. This information allows personalised results to be returned to the correct participant. All answers will be anonymous to the Delphi panel, but not to the research team.

________________________________________________________________

End of Block: Introduction

Start of Block: research activities

Q2.1 Research activities an optional implementation cost
The responses from Round 1 indicated that including research costs as an implementation cost is dependent on the type and reason for the study. For example, research for the purpose of furthering implementation science knowledge may not be relevant when quantifying implementation costs, as these costs would not extend to other institutions or sites considering the implementation of a particular innovation. Conversely, research costs may be relevant to include when conducting quality improvement studies or when the intervention would otherwise not be implemented without local evidence to support its safety, efficacy, or cost-effectiveness, for example.

As a result of this feedback, we have decided to acknowledge research costs as being a potentially relevant implementation cost within the costing instrument’s supporting manuscript. It will be made clear that the relevance of research costs is context-specific and should be determined by the user of the costing instrument.

Please indicate the extent to which you agree/disagree with the following statement.

|  | Disagree | Neutral | Agree |
| --- | --- | --- | --- |

|  | 1 | 2 | 3 | 4 | 5 | 6 | 7 | 8 | 9 | 10 |
| --- | --- | --- | --- | --- | --- | --- | --- | --- | --- | --- |

| The cost of ‘research activities’ should be mentioned as an optional inclusion in the manuscript published alongside the implementation costing instrument, with explanation provided about when these costs may be relevant to include. (Research activities can include preparing study protocols/ ethics applications, recruiting participants, obtaining consent, managing research data, and dissemination of research findings.) () | 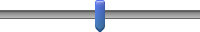 |
| --- | --- |

Q2.2 Please expand on your answer above and explain why you agree/disagree in the space supplied below.

________________________________________________________________

________________________________________________________________

________________________________________________________________

________________________________________________________________

________________________________________________________________

End of Block: research activities

Start of Block: scope

Q3.1 Refinement of instrument scope and removal of supporting appendices

There were mixed responses and a lack of consensus on the content included within the supporting material (Appendix A, B, C) within the first round of the e-Dephi panel, likely due to ambiguity in the scope of the instrument. After reflecting on these responses, the research team decided to refine the overall scope and purpose of the instrument to be more consistent with our original aim to provide practical, user-friendly templates to assist in the collection of appropriate costing data.

We determined that educating users on implementation science theory, frameworks and strategies is beyond the scope of this costing instrument. As such, we have now removed the supporting education-related materials from the costing instrument (Appendix A, B, C). This information will be replaced with appropriate references to key studies within the implementation science literature where users can further their understanding as required. These references will be included within an open access published paper that outlines the development and intended use of the costing instrument. 

We recognise that there is an implicit assumption that the user will have some prior knowledge of implementation science before using the costing instrument, which we believe is reasonable given their intention to use and cost implementation strategies.


We propose that the refined costing instrument contains the following succinct description of scope to clarify its intended use:

*This instrument aims to cost implementation strategies. An implementation strategy is a method or technique used to enhance the adoption, implementation, and sustainability of an under-utilised intervention. This is separate to what may be considered an intervention or technology related cost, which is the evidence-based practice, programme, policy, process, or guideline recommendation that is being implemented (*Pinnock et al., 2017*).*
  

Please indicate the extent to which you agree/disagree with the following statements.

|  | Disagree | Neutral | Agree |
| --- | --- | --- | --- |

|  | 1 | 2 | 3 | 4 | 5 | 6 | 7 | 8 | 9 | 10 |
| --- | --- | --- | --- | --- | --- | --- | --- | --- | --- | --- |

| The refined scope adequately describes the purpose of the costing instrument. () | 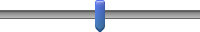 |
| --- | --- |
| The paper cited in the statement summarising the instrument scope is appropriate to describe the difference between implementation and intervention related costs. () | 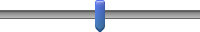 |
| It is appropriate to assume users of the costing instrument will have some level of prior implementation science knowledge. () | 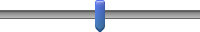 |

Q3.2 Please expand on your answers above and explain why you agree/disagree in the space supplied below.

________________________________________________________________

________________________________________________________________

________________________________________________________________

________________________________________________________________

________________________________________________________________

End of Block: scope

Start of Block: Updated instrument

Q4.1 The updated implementation costing instrument

The updated implementation costing instrument comprises of 3 data collection templates. One template (Table 1) is used to plan and identify the implementation costs. The other two templates (Table 2 and Table 3) are used to collect the data necessary to quantify the implementation costs. Automated summary tables are created when users complete Table 2 and Table 3 in the supplied excel spreadsheet.

     Table 1 updates
The purpose of Table 1 is to help plan what data is required to be collected. This will allow for comprehensive and targeted data collection later in the costing instrument where users will assign monetary values to specific activities and resources.

In Table 1, users will list their pre-selected implementation strategies and outline which activities are needed to operationalise the strategies. The labour and non-labour resources used to deliver each activity are listed in the final column.

In response to the feedback from Round 1, the following updates were made to Table 1:

'Labour' was added as a potential resource.

More examples were provided.

'etc.' was added to indicate that the listed activities are not exhaustive.

The header information was refined.  

Table 1: Planning template.  

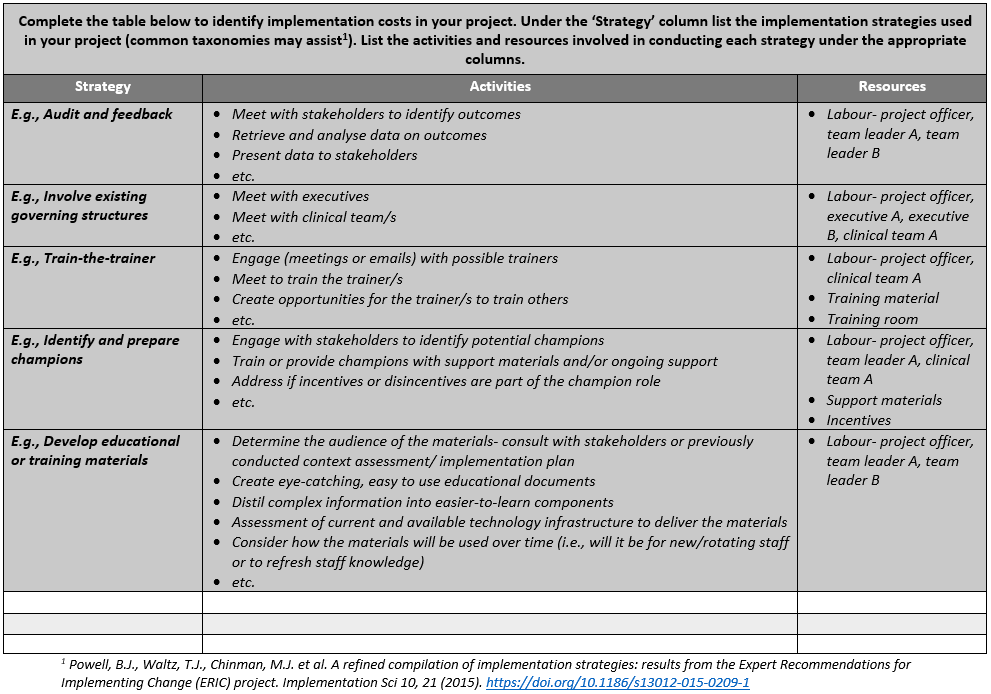


Link for the above cited paper.

  
 Please indicate the extent to which you agree/disagree with the following statements.

|  | Disagree | Neutral | Agree |
| --- | --- | --- | --- |

|  | 1 | 2 | 3 | 4 | 5 | 6 | 7 | 8 | 9 | 10 |
| --- | --- | --- | --- | --- | --- | --- | --- | --- | --- | --- |

| The refined Planning Template (Table 1) remains useful. () | 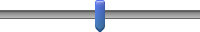 |
| --- | --- |
| The paper cited in the Planning Template (Table 1) is appropriate as a guide for users who would like more information about classification of implementation strategies. () | 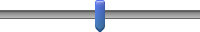 |

Q4.2 Table 2 updates
The purpose of Table 2 is to collect data on labour resources associated with the implementation strategies. This will allow for comprehensive cost summaries to be produced.

Issues relating to accuracy and precision of the data collected within this table were mentioned in Round 1’s feedback, specifically surrounding potential for recall bias and the frequency with which this table should be completed. These are important considerations which will be mentioned in the supporting manuscript, however, the decision on frequency of completion will ultimately remain with the user to determine based on what is appropriate for their project.

In response to other feedback from Round 1, the following updates were made to Table 2:

- Combined the previous ‘activity log template’ and ‘aggregate labour costs template’ into one comprehensive template.
- ‘Outcome’ column changed to ‘Notes’.
- Split ‘Who’ column so that each personnel type/role has their own row.
- Added hourly wage rate and number of personnel involved.
- Removed ‘Date’ column to reduce burden of data collection, as date may not be relevant for many studies given all costs will fall within an implementation phase.
- Removed ‘Resource’ column as is labour resource only.

Table 2: Labour resource data collection template.  

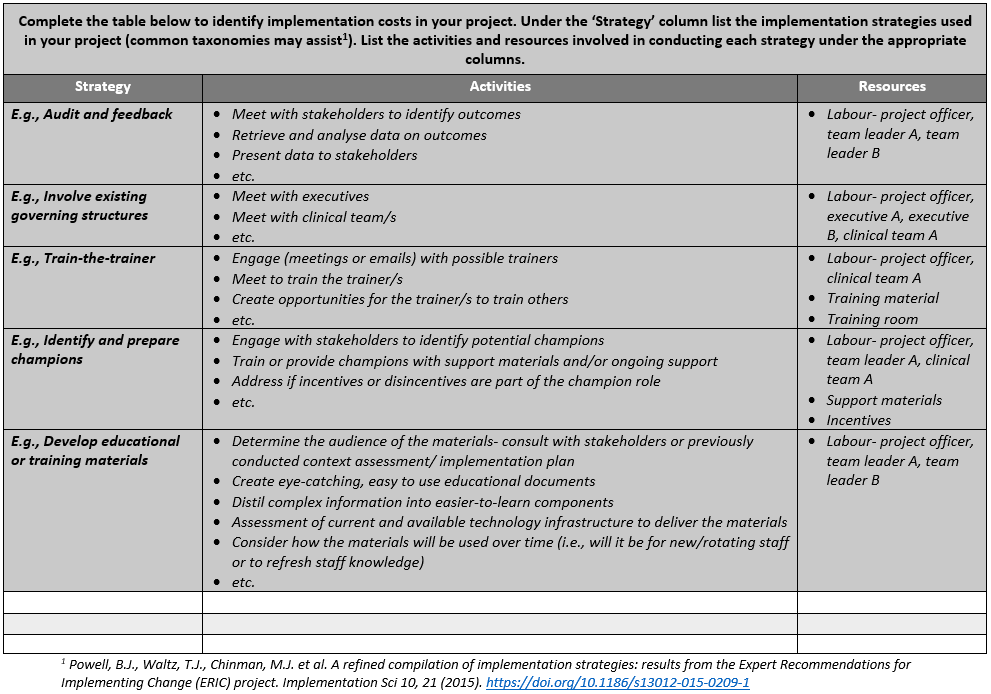


Please indicate the extent to which you agree/disagree with the following statement.

|  | Disagree | Neutral | Agree |
| --- | --- | --- | --- |

|  | 1 | 2 | 3 | 4 | 5 | 6 | 7 | 8 | 9 | 10 |
| --- | --- | --- | --- | --- | --- | --- | --- | --- | --- | --- |

| The refined Labour Data Collection Template (Table 2) remains useful. () | 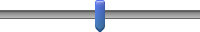 |
| --- | --- |

Q4.3 Table 3 updates
The purpose of Table 3 is to collect the cost data on non-labour resources associated with the implementation strategies. This will allow for comprehensive cost summaries later in the costing instrument.

In response to the feedback from Round 1, the following updates were made to Table 3:

- Added ‘Monetary or opportunity cost’ column.
- Changed ‘resource’ to ‘non-labour resource’ column heading to improve clarity.
- Added more columns on cost source, year, and unit description.
- Another example was added.

Table 3: Non-labour resource data collection template.

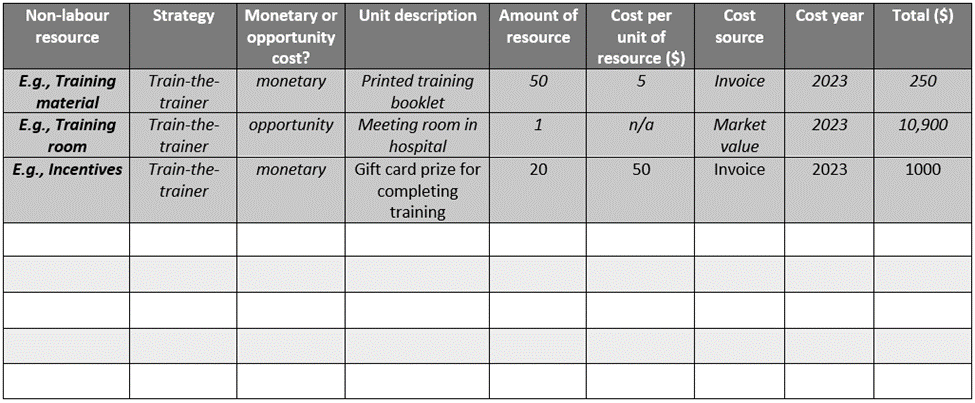


  Please indicate the extent to which you agree/disagree with the following statement.

|  | Disagree | Neutral | Agree |
| --- | --- | --- | --- |

|  | 1 | 2 | 3 | 4 | 5 | 6 | 7 | 8 | 9 | 10 |
| --- | --- | --- | --- | --- | --- | --- | --- | --- | --- | --- |

| The refined Non-Labour Data Collection Template (Table 3) remains useful. () | 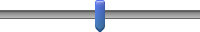 |
| --- | --- |

Q4.4
Please indicate the extent to which you agree/disagree with the following statement. 
If needed, see the previous version implementation costing instrument to review Appendix A, B, C.

|  | Disagree | Neutral | Agree |
| --- | --- | --- | --- |

|  | 1 | 2 | 3 | 4 | 5 | 6 | 7 | 8 | 9 | 10 |
| --- | --- | --- | --- | --- | --- | --- | --- | --- | --- | --- |

| The refined costing instrument could adequately capture implementation costs. () | 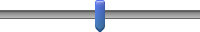 |
| --- | --- |
| The costing instrument remains fit for purpose without the inclusion of supporting materials (Appendix A, B, C). () | 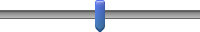 |

Q4.5 Please expand on your answers above and explain why you agree/disagree in the space supplied below.

________________________________________________________________

________________________________________________________________

________________________________________________________________

________________________________________________________________

________________________________________________________________

End of Block: Updated instrument

Start of Block: generalised

Q5.1 Removed specificity to digital health

In Round 1 the costing instrument was proposed to be specific to digital health and the feedback suggested more digital health specific examples would be helpful. Given the refinements in the instrument's scope (previously outlined), the research team have proposed to make the instrument more generic in nature so that it has potential to be applied in other settings. This is because implementation strategies (as opposed to specific interventions or technologies) are inherently generic and transferrable across settings.

The costing instrument's feasibility and generalisability beyond the field of digital health will be explored and tested in future studies.

Please indicate the extent to which you agree/disagree with the following statements.

|  | Disagree | Neutral | Agree |
| --- | --- | --- | --- |

|  | 1 | 2 | 3 | 4 | 5 | 6 | 7 | 8 | 9 | 10 |
| --- | --- | --- | --- | --- | --- | --- | --- | --- | --- | --- |

| The costing instrument should be generic so that it can used outside the field of digital health. () | 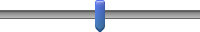 |
| --- | --- |
| The costing instrument could potentially be used outside the field of digital health in its current form. (The back button in this survey can be used to revisit the updated costing instrument). () | 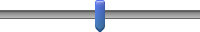 |

Q5.2 Please expand on your answers above and explain why you agree/disagree in the space supplied below.

________________________________________________________________

________________________________________________________________

________________________________________________________________

________________________________________________________________

________________________________________________________________

End of Block: generalised

Start of Block: digital functionality

Q6.1 Improved digital functionality

In response to the feedback from Round 1, the digital functionality of the costing instrument has been improved. Specifically, we have produced an electronic version of the data collection templates (Tables 1, 2 and 3) in MS Excel, including use of ‘drop-down’ options where possible to optimise data quality.

The excel file includes two summary tables that are automatically populated with data entered from the templates. These tables summarise implementation cost subtotals by implementation strategy and by personnel type/role.


 The electronic version can be viewed by downloading this file: Digitised costing instrument.


 Please indicate the extent to which you agree/disagree with the following statement.

|  | Disagree | Neutral | Agree |
| --- | --- | --- | --- |

|  | 1 | 2 | 3 | 4 | 5 | 6 | 7 | 8 | 9 | 10 |
| --- | --- | --- | --- | --- | --- | --- | --- | --- | --- | --- |

| The automated summary tables are a useful addition to the costing instrument. () | 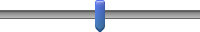 |
| --- | --- |
| The MS Excel version of the costing templates is user-friendly. () | 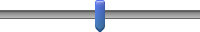 |
| Additional versions of the data collection templates in alternative formats (e.g. RedCap, MS Word, pdf) should be made available. () | 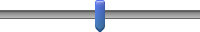 |

Q6.2 Please expand on your answers above and explain why you agree/disagree in the space supplied below.

________________________________________________________________

________________________________________________________________

________________________________________________________________

________________________________________________________________

________________________________________________________________

End of Block: digital functionality

Start of Block: Conclusion

Q7.1

Please indicate the extent to which you agree/disagree with the following statement.

|  | Disagree | Neutral | Agree |
| --- | --- | --- | --- |

|  | 1 | 2 | 3 | 4 | 5 | 6 | 7 | 8 | 9 | 10 |
| --- | --- | --- | --- | --- | --- | --- | --- | --- | --- | --- |

| Overall, the refinements have improved the costing instrument. () | 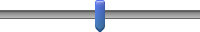 |
| --- | --- |

Q7.2 Please provide any other comments in the space supplied below.

________________________________________________________________

________________________________________________________________

________________________________________________________________

________________________________________________________________

________________________________________________________________

End of Block: Conclusion

**Stimulus Material**


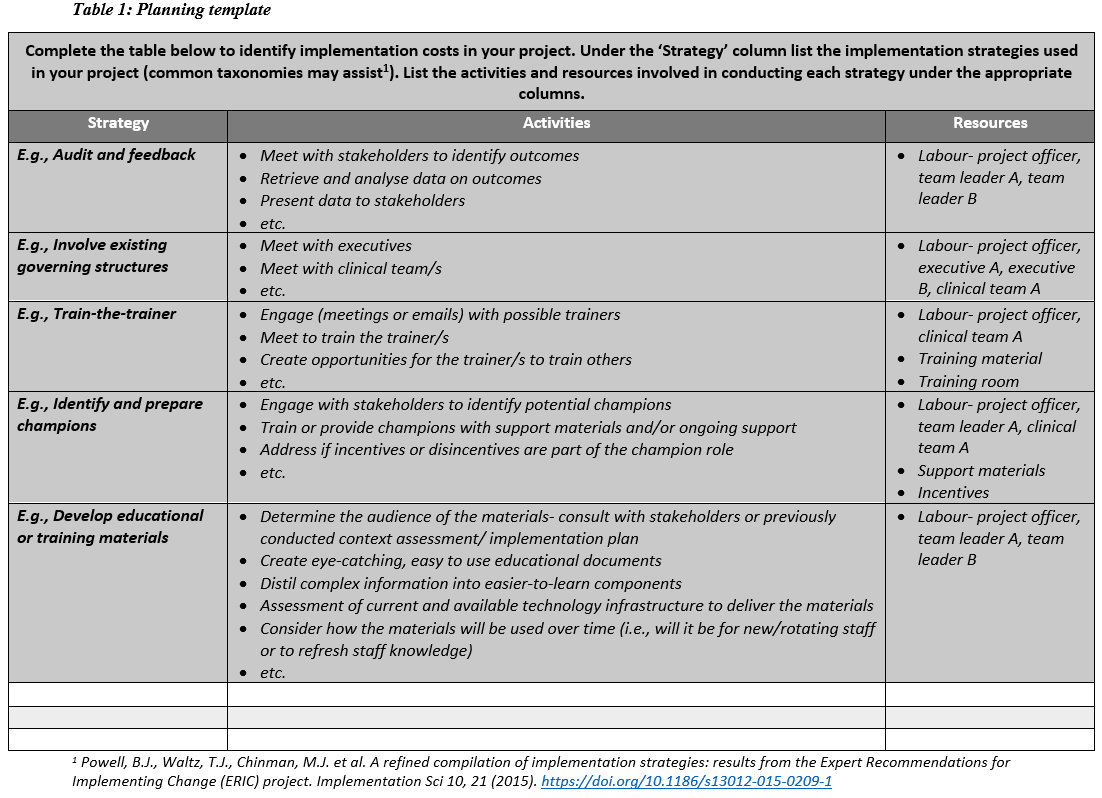


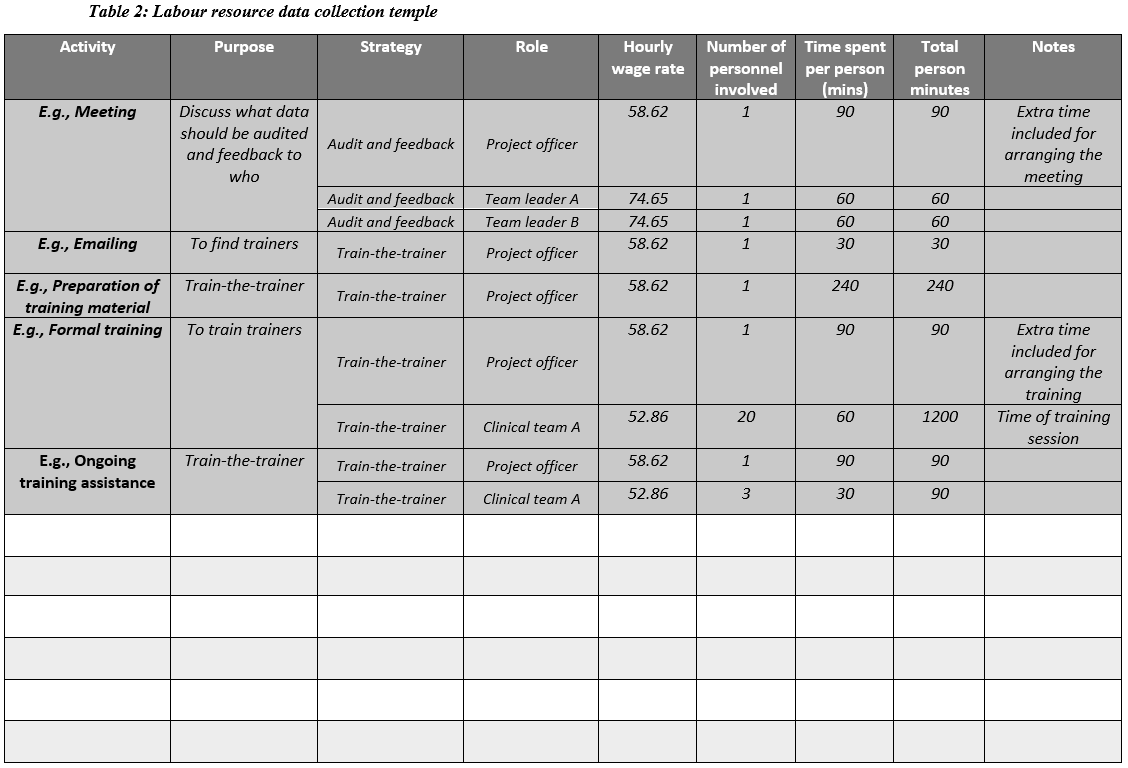


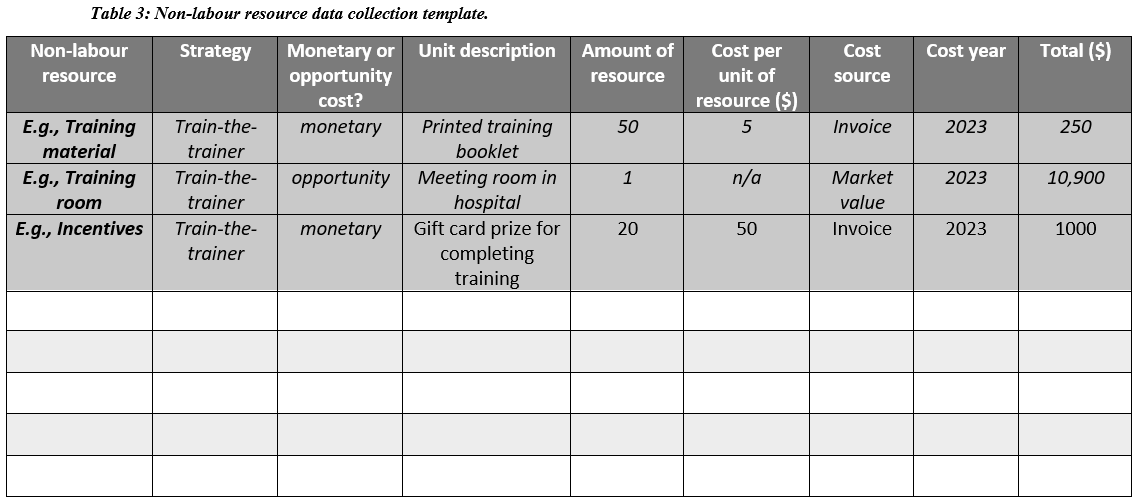

Supplement: Supplementary file 3 — Additional file 3. Questionnaire and stimulus material for Round 2 of e-Delphi. [file 13012_2025_1423_MOESM3_ESM.docx]
