# Supplementary material for: Development of an instrument (Cost-IS) to estimate costs of implementation strategies for digital health solutions: a modified e-Delphi study
Source: Implement Sci. 2025 Mar 7;20:13. doi: 10.1186/s13012-025-01423-w (PMC11889902; doi:10.1186/s13012-025-01423-w)
Supplement: Supplementary file 4 — Additional file 4. Participant characteristics and e-Delphi results. [file 13012_2025_1423_MOESM4_ESM.docx]

**Additional File 4: Participant characteristics and e-Delphi results**

**Table 1. Spread of expertise among participants.**

|  | Implementation Science | Health Economics | Digital Health |
| --- | --- | --- | --- |
| # | 6 | 6 | 7 |
| % | 50% | 50% | 58% |

Implementation

science

**n= 2**

Health

economics

Digital health

**n= 1**

**n= 3**

**n= 1**

**n= 1**

**n= 2**

**n= 2**

**Figure 1. Participants’ experience in implementation science, health economics and digital health.**

**Table 2. Participant characteristics.**

| Characteristic | Count |
| --- | --- |
| Gender | |
| Female | 8 |
| Male | 4 |
| Location | |
| National (Australia) | 10 |
| *Queensland (local)* | *7* |
| *New South Wales* | *2* |
| *South Australia* | *1* |
| International | 2 |
| *United States* | *2* |
| Industry | |
| Academic and Healthcare | 5 |
| Academic only | 6 |
| Government | 1 |

**Table 3. Consensus and agreement percentages from Round 1 and Round 2 questionnaires**

| Round | Question number | Question | Agreement (%) | Consensus (Y/N) |
| --- | --- | --- | --- | --- |
| **Round 1** | | | | |
| 1 | 2.1.1 | It is important to define the implementation process (planning, engaging, executing, reflecting and evaluating) in the instrument's scope. | 92% | Yes |
| 1 | 2.1.2 | The proposed scope (planning, engaging, executing, reflecting and evaluating) adequately captures implementation costs. | 75% | Yes |
| 1 | **2.3.1** | **I believe research activities are an implementation cost, in an implementation study. (Research activities can include preparing study protocols/ ethics applications, recruiting participants, obtaining consent, managing research data, and dissemination of research findings.)** | **42%** | **No** |
| 1 | 3.1.1 | Individuals other than implementation scientists could complete Table 1. | 75% | Yes |
| 1 | 3.1.2 | Table 1 is suitably flexible for use across a range of initiatives. | 83% | Yes |
| 1 | 3.1.3 | Table 1 is practical (i.e., functional in design) if supplied in printable and electronic (e.g., word processing, spreadsheet) formats. | 75% | Yes |
| 1 | 3.1.4 | Table 1 is a useful aid for identifying implementation costs (i.e., has value). | 75% | Yes |
| 1 | 3.3.1 | The listed implementation strategies are comprehensive (i.e., key strategies are present). | 75% | Yes |
| 1 | 3.3.2 | Categorising the implementation strategies by implementation phase is useful (i.e., makes it easier to use). | 83% | Yes |
| 1 | **3.5.1** | **The listed activities and resources are comprehensive (i.e., key ones are present).** | **42%** | **No** |
| 1 | **3.5.2** | **Appendix C is a valuable inclusion in the instrument.** | **50%** | **No** |
| 1 | 4.1.1 | Table 2 can comprehensively collect data on implementation costs (i.e., key data can be captured). | 83% | Yes |
| 1 | 4.1.2 | Table 2 is a useful aid (i.e., has value) to collect data on implementation costs. | 83% | Yes |
| 1 | 5.1.1 | Table 3 can comprehensively aggregate data on implementation labour costs (i.e., key costs can be collated). | 83% | Yes |
| 1 | 5.1.2 | Table 3 is a useful aid (i.e., has value) to aggregate data on implementation labour costs. | 75% | Yes |
| 1 | 5.3.1 | Table 4 can comprehensively aggregate data on implementation resource costs (i.e., key costs can be collated). | 75% | Yes |
| 1 | 5.3.2 | Table 4 is a useful aid (i.e., has value) to aggregate data on implementation resource costs. | 75% | Yes |
| **Round 2** | | | | |
| 2 | 2.1.1 | The cost of ‘research activities’ should be mentioned as an optional inclusion in the manuscript published alongside the implementation costing instrument, with explanation provided about when these costs may be relevant to include. (Research activities can include preparing study protocols/ ethics applications, recruiting participants, obtaining consent, managing research data, and dissemination of research findings.) | 100% | Yes |
| 2 | 3.1.1 | The refined scope adequately describes the purpose of the costing instrument. | 92% | Yes |
| 2 | 3.1.2 | The paper cited in the statement summarising the instrument scope is appropriate to describe the difference between implementation and intervention related costs. | 92% | Yes |
| 2 | **3.1.3** | **It is appropriate to assume users of the costing instrument will have some level of prior implementation science knowledge.** | **67%** | **No** |
| 2 | 4.1.1 | The refined Planning Template (Table 1) remains useful. | 100% | Yes |
| 2 | 4.1.2 | The paper cited in the Planning Template (Table 1) is appropriate as a guide for users who would like more information about classification of implementation strategies. | 100% | Yes |
| 2 | 4.2.1 | The refined Labour Data Collection Template (Table 2) remains useful. | 92% | Yes |
| 2 | 4.2.2 | The refined Non-Labour Data Collection Template (Table 3) remains useful. | 92% | Yes |
| 2 | 4.4.1 | The refined costing instrument could adequately capture implementation costs | 83% | Yes |
| 2 | 4.4.2 | The costing instrument remains fit for purpose without the inclusion of supporting materials (Appendix A, B, C). | 83% | Yes |
| 2 | 5.1.1 | The costing instrument should be generic so that it can used outside the field of digital health. | 75% | Yes |
| 2 | **5.1.2** | **The costing instrument could potentially be used outside the field of digital health in its current form.** | **67%** | **No** |
| 2 | 6.1.1 | The automated summary tables are a useful addition to the costing instrument. | 92% | Yes |
| 2 | 6.1.2 | The MS Excel version of the costing templates is user-friendly | 92% | Yes |
| 2 | **6.1.3** | **Additional versions of the data collection templates in alternative formats (e.g. RedCap, MS Word, pdf) should be made available.** | **33%** | **No** |
| 2 | 7.1.1 | Overall, the refinements have improved the costing instrument. | 92% | Yes |

**Table 4. Summary of the updates to the costing instrument as a result of the e-Delphi process**

| **Instrument section** | **Updates made included** |
| --- | --- |
| Template 1 | - 'Labour' was added as a potential resource. - More examples were provided. - 'etc.' was added to indicate that the listed activities are not exhaustive. - The header information was refined. |
| Template 2A | - Combined the previous ‘activity log template’ and ‘aggregate labour costs template’ into one comprehensive template. - ‘Outcome’ column changed to ‘Notes’. - Split ‘Who’ column so that each personnel type/role has their own row. - Added hourly wage rate and number of personnel involved. - Removed ‘Date’ column to reduce burden of data collection, as date may not be relevant for many studies given all costs will fall within an implementation phase. - Removed ‘Resource’ column as is labour resource only. |
| Template 2B | - Added ‘Monetary or opportunity cost’ column. - Changed ‘resource’ to ‘non-labour resource’ column heading to improve clarity. - Added more columns on cost source, year, and unit description. - Another example was added. |
